# Transferrin and octaarginine modified dual-functional liposomes with improved cancer cell targeting and enhanced intracellular delivery for the treatment of ovarian cancer

**DOI:** 10.1080/10717544.2018.1435747

**Published:** 2018-02-12

**Authors:** Pranali Deshpande, Aditi Jhaveri, Bhushan Pattni, Swati Biswas, Vladimir Torchilin

**Affiliations:** a Center for Pharmaceutical Biotechnology and Nanomedicine, Northeastern University, Boston, MA, USA;; b Department of Pharmacy, Birla Institute of Technology & Science-Pilani, Hyderabad Campus, Hyderabad, India

**Keywords:** Liposomes, transferrin, doxorubicin, octaarginine, active targeting, ovarian cancer, TfR over-expression, dual-functional liposomes

## Abstract

Off-target effects of drugs severely limit cancer therapy. Targeted nanocarriers are promising to enhance the delivery of therapeutics to tumors. Among many approaches for active tumor-targeting, arginine-rich cell penetrating peptides (AR-CPP) and ligands specific to target over-expressed receptors on cancer-cell surfaces, are popular. Earlier, we showed that the attachment of an AR-CPP octaarginine (R8) to the surface of DOXIL^®^ (Doxorubicin encapsulated PEGylated liposomes) improved cytoplasmic and nuclear DOX delivery that enhanced the cytotoxic effect *in vitro* and improved therapeutic efficacy *in vivo*. Here, we report on DOX-loaded liposomes, surface-modified with, R8 and transferrin (Tf) (Dual DOX-L), to improve targeting of A2780 ovarian carcinoma cells via the over-expressed transferrin receptors (TfRs) with R8-mediated intracellular DOX delivery. Flow cytometry analysis with fluorescently labeled DualL (without DOX) showed two-fold higher cancer-cell association than other treatments after 4 h treatment. Blocking entry pathways of R8 (macropinocytosis) and Tf (receptor-mediated endocytosis, RME) resulted in a decreased cancer-cell association of DualL. Confocal microscopy confirmed involvement of both entry pathways and cytoplasmic liposome accumulation with nuclear DOX delivery for Dual DOX-L. Dual DOX-L exhibited enhanced cytotoxicity *in vitro* and was most effective in controlling tumor growth *in vivo* in an A2780 ovarian xenograft model compared to other treatments. A pilot biodistribution study showed improved DOX accumulation in tumors after Dual DOX-L treatment. All results collectively presented a clear advantage of the R8 and Tf combination to elevate the therapeutic potential of DOX-L by exploiting TfR over-expression imparting specificity followed by endosomal escape and intracellular delivery via R8.

## Introduction

This year, in the US alone, the American Cancer Society has predicted 4630 cancer-cases each day, of which, approximately 1650 will result in death. These alarming numbers indicate that cancer remains a leading cause of death showing an upward trend (Magadala et al., 2008). Traditional chemotherapy drug candidates still continue to remain a popular choice of treatment in the clinic, either alone or in combination for second-line therapy at advanced stages (Ohe et al., 2007). Despite promising therapeutic benefits these drugs have limitations that include a narrow therapeutic window, poor pharmacokinetics, low solubility, rapid clearance from the circulation, poor bioavailability and multi-drug resistance, leading to recurrence and harmful off-target effects. Advances in the understanding of cancer biology, the tumor microenvironment and key players in tumor progression, have resulted in remarkable progress in cancer prevention, treatment and management (Hanahan & Weinberg, 2011; Jain, 2013). Nanomedicine platforms like liposomes offer benefits that can overcome the challenges associated with traditional chemotherapy and have helped transform promising drug candidates into stable and efficient cancer therapies (Torchilin et al., 1994; Gabizon et al., 2003; Aslan et al., 2013; Zhao et al., 2013). Liposome formulations can take advantage of the leaky tumor vasculature, escape the circulation through the abnormal endothelial junction gaps and accumulate in the tumor tissue by exploiting its dysfunctional lymphatic drainage (Duggan & Keating, 2011; Kumari et al., 2016). This phenomenon has been well studied upon initial work by Maeda and colleagues in the 1980s, suggesting preferential accumulation of liposomes in tumors with an altered microenvironment, compared to healthy tissues that have tighter endothelial junctions, ultimately controlling the toxicity to healthy tissues boosting their safety profile (Aslan et al., 2013). This attribute of liposomes to accumulate in tumor tissues, as a virtue of their small size is known as passive targeting via the ‘enhanced permeability and retention effect’ (EPR effect) (Torchilin, 2006, 2010).

Doxorubicin (DOX) is one of the most popular anthracycline anti-cancer drugs. DOX kills tumor cells via many nuclear events that lead to DNA damage, apoptosis and ultimately cell death (Minotti et al., 2004). It has been successfully used in the clinic but is often associated with severe side-effects like cardiotoxicity even at therapeutic doses (O’Brien et al., 2004; Peng et al., 2005; Octavia et al., 2012). DOX-encapsulating nanocarriers overcome the issue of free drug toxicity and have become extremely popular for application in metastatic, breast and ovarian cancer (Judson et al., 2001; Duggan & Keating, 2011). DOXIL^®^ (DOX-encapsulated liposomes) an early prototype, helps to minimize toxic effects of DOX and takes advantage of passive targeting into tumors via the EPR effect. However, DOXIL^®^ shows poor penetration into the tumor tissue (Duggan & Keating, 2011). Sole reliance on an EPR effect to get the drug-encapsulated liposomes to the tumors is insufficient due, in part, to heterogeneities in the vasculature. This has led to emphasis on ‘active targeting techniques’ to modify the liposome surface with ligands that can recognize cancer cells, guide the drug-encapsulated liposomes to the tumor tissues and promote intra-cellular delivery of the therapeutic cargo (Magadala et al., 2008; Li et al., 2009; Sawant & Torchilin, 2010; Kolhatkar et al., 2011; Mehra et al., 2013; Nogueira et al., 2015).

Arginine-rich cell-penetrating peptides (CPPs) are a popular class of penetration enhancers used for nanoparticle drug delivery systems. TATp one of the most researched CPPs is derived from the 86-mer trans-activating transcriptional activator (TAT protein), that is encoded by the human immunodeficiency virus type-1 (HIV-1). Polyarginines resemble the translocation domain in TATp, that has made them a popular choice as a cell penetration tool (Frankel & Pabo, 1988, Vives et al., 1997, Futaki et al., 2001, Torchilin et al., 2003, Gupta et al., 2005, Futaki et al., 2007, Torchilin 2008, Sawant & Torchilin, 2010, Schmidt et al., 2010). The optimum chain length for efficient translocation for polyarginines is considered to be 8 arginine units (R8). A great deal of discussion has been dedicated to the uptake mechanisms involved with R8. However, the consensus is that the preferred mode of cell entry is via macropinocytosis (Khalil et al., 2006, Futaki et al., 2007; El-Sayed et al., 2009; Futaki et al., 2013). R8 has been successfully reported in a number of studies to enhance intracellular delivery of therapeutic cargo of nanoparticle drug delivery systems (Khalil et al., 2011; Koshkaryev et al., 2013).

The human transferrin receptor (TfR) is a transmembrane single-chain glycoprotein comprised of 700 amino acids with two disulfide subunits and is responsible for the transport of ferric ions (Cheng et al., 2004; Kakudo et al., 2004; Kobayashi et al., 2007). Transferrin (Tf) is a serum glycoprotein with a MW of 80 KDa and acts as a carrier of ferric ions into cells via the TfR. Cancer cells need more iron compared to normal cells to keep up with their rapid proliferation rates (Kobayashi et al., 2007; Daniels et al., 2012). This effect leads to over-expression of TfRs on certain types of tumor cells, where the high turnover rate of receptors is directly proportional to the proliferation potential of the tumor and correlates with the progression of the disease (Gatter et al., 1983; Hogemann-Savellano et al., 2003). Elevated receptor expression can be exploited for active targeting of therapeutic cargo-loaded nanoparticles such as liposomes to cancer cells. Tf-targeted nanocarriers have been reported to improve specificity of the drug cargo towards cancer cells via receptor-mediated endocytosis (RME) (Choi et al., 2010; Koshkaryev et al., 2012). Many studies have combined Tf with PEG on PEGylated liposomes to achieve targetability and longevity for drug delivery to solid tumors (Torchilin, 2006; Li et al., 2009).

Previously, we reported that R8-DOXIL^®^ improved intracellular and nuclear DOX delivery to cancer cells by helping the liposomes to overcome the cell membrane barrier and capture by endosomes (Biswas et al., 2013). Endosomal escape of R8-DOXIL^®^ followed by nuclear DOX delivery translated in a more pronounced cytotoxic effect both in *vitro* and *in vivo* compared to non-modified DOXIL^®^.

Since R8 is nonselective towards cancer cells, in our current study we have explored the development of dual-functional liposomes (DualL) modified with both Tf and R8, to enhance selectivity towards ovarian cancer cells. A targeted liposome (LP) delivery system with dual moieties, arginine-glycine-aspartic acid peptide (RGD) and Tf to deliver Paclitaxel (PTX) for glioma therapy is successfully relevant, reinforcing the use of dual functionalities where the authors showed greatest antitumor effects *in vivo* for the PTX-loaded RGD/TF-LP (Qin et al., 2014). Considering that the reports on dual-targeted systems with Tf and CPP, in ovarian cancer, are limited, we hypothesized that surface-modification of DOX-loaded liposomes with R8 and Tf (Dual DOX-L), will improve selectively of the liposomes toward the over-expressed TfRs and help in better cyotosolic DOX delivery leading to enhanced anti-cancer effects both *in vitro* and *in vivo*.

## Materials

Hydrogenated soy phosphatidylcholine (HSPC), *N*-(carbonyl-methoxypolyethylene glycol 2000)-1, 2-distearoyl-sn-glycero-3-phosphoethanolamine sodium salt (mPEG-DSPE), and cholesterol were purchased from Avanti Polar Lipids (Alabaster, AL, USA). 1, 2-distearoyl-*sn*-glycero-3-phosphoethanolamine-*N*-[methoxy (polyethylene glycol)-2000] (ammonium salt) (PEG_2K_-DOPE), 1, 2-Dioleoyl-sn-glycero-3-phosphoethanolamine (DOPE) was from Avanti Polar Lipids (AL, USA). Octa-arginine peptide (RRRRRRRR, M.W. 1267.46 Da) was synthesized by the Tufts University Core Facility (Boston, MA, USA). Human holo-Transferrin (Tf) was purchased from Sigma. PEG-(pNP)_2_ (Polyoxyethylene-bis (p-nitrophenyl carbonate) (MW 3500-NPC-PEG_3.4K_-NPC) and (MW 2000-NPC-PEG_2K_-NPC) were obtained from Laysan Bio (AL, USA). Sepharose CL4-B was purchased from Bio-Rad. Transferrin-Alexa Fluor 488, Transferrin-Alexa Fluor 680 and Hoechst 33342 were purchased from Molecular Probes (Eugene, OR, USA). Fluorescein isothiocyanate-dextran (FITC-Dextran 70 KDa) and Amiloride hydrochloride hydrate were purchased from Sigma-Aldrich. DiO′; DiOC_18_ (3) (3,3′-Dioctadecyloxacarbocyanine Perchlorate) green dye was purchased from Invitrogen (Thermo Scientific). *para*-formaldehyde was from Electron Microscopy Sciences (Hatfield, PA, USA). Fluoromount-G was from Southern Biotech (Birmingham, AL, USA). Doxorubicin hydrochloride was purchased from LC laboratories (Woburn, MA, USA). The Cell Titer-Blue^®^ Cell Viability Assay was purchased from Promega (Madison, WI, USA). All other chemicals and buffer components were analytical grade preparations.

### Cell lines

The human ovarian carcinoma cell line (A2780), normal mouse fibroblasts (NIH-3T3), normal human skin fibroblasts (CCD 27 SK) and rat cardiomyocytes (H9C2) were purchased from the American Type Culture Collection (Manassas, VA). Dulbecco’s modified Eagle’s media (DMEM) and heat-inactivated fetal bovine serum (FBS) was obtained from Gibco (Carlsbad, CA, USA). Concentrated penicillin/streptomycin stock solution was from CellGro^®^ (Herndon, VA, USA). All other chemicals and solvents were of analytical grade, purchased from Sigma-Aldrich and used without further purifications. The cells were grown in DMEM with 2 mM l-glutamine, supplemented with 10% (v/v) heat-inactivated FBS, 100 units/mL penicillin G and 100 µg/mL streptomycin. Cultures were maintained in a humidified atmosphere at 37 °C and 5% CO_2_.

### Animals

Immunodeficient female Ncr NU/NU nude mice (4–6 weeks old) were purchased from Taconic Biosciences, Hudson, New York, NY, USA. All animal procedures were performed according to an animal care protocol approved by Northeastern University Institutional Animal Care and Use Committee. Mice were housed in groups of 5 at 19–23 °C with a 12 h light-dark cycle and allowed free access to food and water.

## Methods

### Preparation of single-ligand and dual-ligand modified liposomes

#### Synthesis of R8-PEG_2k_-PE and tf-PEG_3.4k_PE polymers

The first step to prepare DualL, was to attach R8 and Tf to the distal tips of PEG blocks via *p*-nitrophenylcarbonyl (pNP) groups (using pNP-PEG_2000_-PE and pNP-PEG_3400_-PE conjugate) to form R8-PEG_2000_-PE and Tf-PEG_3400_-PE conjugate, respectively. To achieve this pNP-PEG_2000_-PE and pNP-PEG_3400_-PE were synthesized and purified according to previously established protocol in our laboratory (Torchilin et al., 2001). Briefly, DOPE was mixed with PEG-(pNP) _2_ (five-fold molar excess) in chloroform followed by dropwise addition of triethylamine (TEA) and stirred overnight at room temperature. The following day, once the organic solvents were evaporated the products were freeze-dried (Labconco, Freeze dry system, Freezone).The resultant pNP-PEG_2000_-PE or pNP-PEG_3400_-PE micelles in HCl (0.01 M) solution were separated from free PEG and pNP on a sepharose CL-4B column. The products obtained were freeze-dried and stored in chloroform at −80 °C.

R8-PEG_2000_-PE was synthesized following a protocol used previously (Biswas et al., 2013). Here, R8 (7.4 mg, 5.86 µmol) and TEA (10 µL) dissolved in 200 µL Dimethyl formamide (DMF) and mixed with a solution of pNP-PEG_2K_-DOPE (10 mg, 3.9 µmol) in chloroform (1.0 mL). The reaction was stirred overnight at room temperature. The chloroform was evaporated and the product was freeze-dried. The dried reaction mixture was dissolved in PBS, pH 8.4, and stirred at room temperature for 4 h followed by dialysis against water using a cellulose ester membrane (MWCO. 2000 Da) overnight. The dialysate was freeze-dried and the solid white fluffy product was dissolved in methanol at 5 mg/mL and stored at −80 °C.

Tf-PEG_3400_-PE was synthesized following a previously well-established protocol (Sawant et al., 2013). A dry lipid film of PNP-PEG_3400_-PE (2 mg) was obtained by evaporation of organic solvents followed by freeze-drying. The dry film was hydrated with 5 mM citrate-buffered saline, pH 5.0, followed by addition of a Tf solution (18 mg Tf in PBS, pH 8.5) with pNP-PEG_3400_-PE in 2× molar excess over Tf. The pH was adjusted to 8.0–8.5 and the reaction was stirred continuously overnight at room temperature. The following day, micelles were dialyzed against 4 L of 10 mM PBS, pH 7.4 using cellulose ester membranes (MWCO 300 KDa). The amount of transferrin in the Tf-PEG_3400_-PE conjugate was estimated by a bicinchoninic acid (BCA) protein assay with pure bovine serum albumin (BSA) as a standard.

#### Formulation of rhodamine-labeled liposomes and DOX-loaded liposomes

To assess the cell association of DualL with cancer cells and their subsequent internalization, Rhodamine-PE (Rh-PE) – labeled liposomes were used initially. Liposomes were prepared by the ammonium sulfate, pH gradient method similar to that of DOXIL^®^ (Gabizon et al., 2003). Briefly, a dry lipid film was obtained from a mixture of hydrogenated soy PC, cholesterol, methoxy DSPE and rhodamine-PE (HSPC:Chol:mPEG-DSPE:RhPE =59:38.21:2:1) (Table S1), by evaporation of the chloroform solution of the combined ingredients, followed by freeze drying for at least 2 h. The dry lipid film was hydrated with 300 mM ammonium sulfate solution, pH 5.5, at a lipid concentration of 15 mg/ml. The hydrated mixture was vigorously vortexed for 5 min to make multilamellar vesicles and then heated in a water bath at 65  °C for about an hour with intermittent vortexing. This solution was then extruded 21 times through a 200 nm pore sized polycarbonate membrane filter (Avanti Polar Lipids, Alabaster, AL, USA) to yield liposomes of uniform particle size. These liposomes were then subjected to desalting columns (Zeba spin desalting columns and devices 7 K MWCO, Thermo-scientific) or alternately dialyzed first against water (3 h) and then 0.9% NaCl overnight (Haran et al., 1993; Mayer et al., 1999; Bajelan et al., 2012; Nie et al., 2012; Jain et al., 2014), to remove unincorporated materials as well as to replace the external phase of the liposomes (buffer exchange) with HBS pH 7.4 to establish a pH gradient (2–3 h) (Gabizon et al., 2003) (Figure S1). For all the cell interaction studies, the fluorescent probe, Rh–PE was added to the liposomes at 1 mol% of the total lipid concentration during liposome preparation.

For DOX-loaded liposomes, a dry lipid film was obtained from a mixture of hydrogenated soy PC, cholesterol, methoxy-PEG-DSPE (HSPC:Chol:mPEG-DSPE= 60:38.21:2) (Table S3). Subsequent steps were the same as mentioned for Rh-labeled liposomes up to the point of dialysis against 0.9% NaCl overnight. At this stage, a free DOX HCl solution was added to the liposomes and incubated at 65 °C for 2 h. The resultant liposomes were subjected to dialysis using a 10,000 MWCO cellulose ester membrane against HBS, pH 7.4, for 2–3 h at 4 °C to remove unincorporated free drug (Figure S7). The amount of DOX loaded within the liposomes was estimated using a Synergy HT multi-detection microplate reader (Biotek, Winooski, VT, USA) by measuring the fluorescence of encapsulated DOX, at wavelengths of 485 nm (excitation) and 590 nm (emission), before and after dialysis, using methanol to break the liposomes.

#### Modification of liposomes with R8-PEG_2k_-PE and tf-PEG_3.4k_PE

Prepared Rh-labeled liposomes and DOX-loaded liposomes were surface-modified with either R8-PEG_2k_-PE or Tf-PEG_3.4k_PE or both via the PEG-PE spacer attached to both these ligands using the post-insertion method (Allen et al., 2002; Sawant et al., 2013). First, Rh-labeled liposomes were modified with either PEG_2K_-DSPE to give Plain liposomes (PL) or R8-PEG_2K_-PE to yield R8 liposomes (R8L). Similarly, DOX-loaded liposomes were modified with either PEG_2K_-DSPE to prepare Plain DOX liposomes (PLDOX-L) or R8-PEG_2000_-PE to prepare R8 DOX liposomes (R8DOX-L). Briefly, Rh-labeled and DOX-loaded liposomes were added to the dry lipid film of PEG_2K_-DSPE (0.90 mg, 0.45 µmol) or R8-PEG_2K_-DOPE (1.64 mg, 0.45 µmol), vortexed for 5 min and stirred overnight at 4 °C for complete hydration of the lipid film (Supplementary data). The concentration of the R8-PEG_2K_-PE co-polymer was optimized at 2 mol % of the total lipid composition of liposomes after conducting an initial investigation (Figure S3). Next, Tf-PEG_3.4K_-PE conjugate was added to both, PL to make Tf liposomes (TfL) and to R8L to make DualL for Tf conjugation at a concentration equivalent to 0.5 mol% (Supplementary data). Similarly, PLDOX-L were incubated with Tf-PEG3400-PE to prepare Tf DOX-loaded liposomes (Tf DOX-L) and R8DOX-L were incubated with Tf–PEG_3.4 K_-PE to prepare dual-functional DOX-loaded liposomes (Dual DOX-L). In brief, the Tf-PEG_3.4K_-PE conjugate was incubated with the liposomes at 37 °C overnight on a shaker to carry of post-insertion of Tf in the liposome bilayer. Ligand density of Tf-PEG_3400_-PE was set to 0.5 mol% Tf after optimization based on cancer-cell interaction (Figure S2).

### Characterization of liposomes

#### Liposome size and zeta potential

The liposome size was determined by the dynamic light scattering using a Coulter N4 MD submicron particle analyzer (Beckman Coulter, Inc., Fullerton, CA, USA). Scattered light was detected at 25 °C at an angle of 90°. For size determination, 10 µl of the liposome samples were diluted in 1.6 ml of HBS buffer, pH 7.4, and measured immediately after preparation. Zeta potential of the liposomal preparations was determined using a ZETA PALS System, Brookhaven Corporation (Holtsville, NY, USA). For zeta potential analysis, 10 µl of the liposome samples were diluted with 1.6 ml of 1 mM KCl solution and analyzed immediately. TEM analysis was used to confirm the size and surface morphology of liposomes using a JEOL JEM-1010 transmission electron microscope (JEOL USA, Inc., Peabody, MA, USA). Ten μl of the liposome samples were added as a drop onto a copper grid with a formvar and carbon coating. Phosphotungstic acid, 1.5% (PTA) was used to negatively stain these samples. Samples were air-dried at room temperature and imaged under a transmission electron microscope operating at an acceleration voltage of 80 kV.

To estimate the stability of the liposomes, the particle size and zeta potential were measured and recorded on day 1 and day 15 post-preparation after storage at 4 °C for all liposome formulations (Table S4).

### Measurement of the percentage of doxorubicin loaded in liposomes

To estimate the concentration of DOX to be used in *in vitro* and *in vivo* studies, the amount of DOX encapsulated inside the liposomes was determined. The DOX-loaded liposomes were dialyzed against HBS, pH 7.4, to remove all unincorporated drug. A before and after dialysis aliquot of liposomes was taken and diluted in methanol to break the liposomes and release encapsulated drug measured by fluorescence detection using a Synergy HT multi-detection microplate reader (Biotek, Winooski, VT, USA) at wavelengths of 485 nm (excitation) and 590 nm (emission). All samples were analyzed in triplicate. The drug loading was determined each time a fresh batch of DOX-loaded liposomes was made, using a standard curve (Figure S8) of known concentration of free DOX in methanol obtained under the same conditions. The loading was determined as follows:

% DOX loaded = amount of DOX obtained in post-dialysis liposome sample ×100

Amount of DOX present in pre-dialysis liposome sample

### *In vitro* studies

#### Cell association of rhodamine-labeled dual-functional liposomes

The cell association of the DualL with cancer cells was assessed and compared to PL, R8L and TfL liposomes by flow cytometry analysis. A2780 cells were allowed to grow until 80% confluence in a T75 flask and after a couple of passages, 0.3–0.5 × 10^6^ cells per well were seeded in 12 well-plates. After overnight incubation, the cells were treated with PL, TfL, R8L or DualL at a dose of 0.1 mg of total lipids per ml of serum free medium for 1 and 4 h incubation periods. The media was removed after the incubation period was completed and the cells were washed with ice-cold PBS, pH 7.4 two to three times to remove free formulation. The cells were then detached using trypsin, followed by deactivation with serum. The cells were then washed again with PBS and centrifuged at 1000 rpm for 5 min. The cell pellet was ultimately re-suspended in PBS pH 7.4 before reading the samples for rhodamine fluorescence using a BD FACS Calibur flow cytometer. The cells were gated using forward (FSC-H)-versus side-scatter (SSC-H) to exclude debris and dead cells before analysis of 10,000 cell counts. Non-cancer cells NIH3T3 cells, H9C2 cells and CCD27SK cells were also tested the using above protocol to assess the association of DualL with non-cancer cells (Figure S5).

#### Effect of macropinocytosis inhibitor on cell association of dual-functional liposomes

Despite a lot of speculation, it has been established that R8 enters the cells by a process of macropinocytosis (Khalil et al., 2006). In order to confirm the involvement of the macropinocytosis pathway in the association and internalization of DualL by cells, the cells were incubated with or without amiloride (5 mM) for 30 min to block macropinocytosis prior to the addition of the formulation. The liposomes were added and incubated with the cells for 4 h in serum-free media. Amiloride (5 mM) was incubated with the cells throughout the experiment. The effect on cell association was studied using FACS by counting 10,000 cells as mentioned previously.

#### Analysis of transferrin receptor-mediated endocytosis of dual-functional liposomes

To examine the contribution of Tf-targeting via TfR endocytic pathway to the uptake of DualL, the competitive inhibition of TfL and DualL was studied in the presence of excess free human transferrin. Holo-Tf was added in serum-free media at a concentration of 2 mg/mL before treatment with liposomes. Here, the cells were incubated with or without free Tf for about 15 min, before treatment and incubation with liposomes for 4 h. The excess Tf was incubated with the cells throughout the experiment. The effect on cell association was observed via flow cytometry using the same procedure mentioned in the previous section.

#### Cellular internalization of dual-functional liposomes by cancer cells using confocal microscopy

To evaluate the mode of entry of the liposomes inside A2780 cancer cells, a study was designed where markers for both receptor-mediated endocytosis and macropinocytosis were used. Tf-Alexa 680 was used as a marker of RME while, FITC dextran 70 KDa was used as a marker for macropinocytosis and Hoechst 3342 was used as a nuclear marker. Rhodamine-PE in the liposome bilayer was considered a marker for liposome localization indicated. A2780 cells were grown to 75–80% confluence on 22 mm coverslips in 12-well cell culture plates. After 24 h the well media were replaced with DMEM supplemented with FITC dextran 70 KDa (0.35 mg/ml) for 30 min. The wells were subsequently washed two to three times with PBS followed by the addition of the formulation. Cells were exposed to 0.1 mg/ml lipid concentration of Rh-PE labeled PL, TfL, R8L or DualL in serum-free media for 4 h. Fifteen minutes before the end of incubation the cells were stained with both, Hoechst 33342 (5 μg/mL) and Tf-Alexa 680 (22.5 μg/ml or 10 μg/ml) in the incubator at 37 degree C. At the end of the incubation, the dye containing medium was removed and cells were washed with ice-cold PBS pH 7.4, three to four times to prevent further internalization of the liposomes attached to the cell-surface. The cells were then fixed with a 4% *p*-formaldehyde solution for 10 min at room temperature. The cells on the cover-slips were mounted with Fluoromount-G medium, examined on a Zeiss Confocal Laser Scanning Microscope (Zeiss LSM 700) equipped with UV (Ex/Em. 385/470 nm), FITC (Ex/Em. 548/595 nm) and a rhodamine filter (Ex/Em. 548/719 nm) for imaging. The images were analyzed using the Image J software version 1.43 (NIH, Bethesda, MD, USA). Pearson’s correlation coefficient (PCC) and Mander’s correlation coefficient (MCC) were calculated using ImageJ to determine color channel overlaps.

#### Cellular internalization of DOX assessed by confocal microscopy

To confirm intracellular DOX delivery another confocal study was performed. Here, the liposome bilayer was labeled with the 1 mol% green fluorescent dye “DiO”. The nuclei were stained using Hoechst and the inherent red fluorescence of DOX was used to assess the cytoplasmic delivery. Briefly, A2780 cells were grown to 75–80% confluence on 22 mm coverslips in 12-well cell culture plates. Post 24 h, the cells were exposed to 0.1 mg/ml lipid concentrations of PL DOX-L, TfL DOX-L, R8 DOX-L and Dual DOX-L in serum-free media for 4 h. Fifteen minutes prior to the end of incubation, the cells were stained with Hoechst 33342 (5 μg/mL) for 15 min in the incubator. At the end of the incubation, the cells were washed with ice-cold PBS, pH 7.4, three to four times and fixed with a 4% *p*-formaldehyde solution for 10 min at room temperature. The cells on the cover-slips were mounted on slides using fluormount-G and analyzed using the confocal microscope as mentioned above.

#### *In vitro* cytotoxicity studies

A2780 cancer cells and non-cancer NIH3T3 cells, H9C2 cells and CCD27SK cells were seeded in 96-well plates at densities of 5000 cells/well for 24 h incubation and 3000 cells/well for 48 h. After 24 h incubation in 5% CO_2_ at 37 °C, the cells were incubated with PL DOX-L, Tf DOX-L, R8 DOX-L and Dual DOX-L at a DOX concentration range of 0.2 to 75 µM for 15 min in serum-free media. After 15 min, the treatment media was removed, cells were washed with fresh media and supplemented with100 μL complete media to further incubate for 24 or 48 h. After incubation, the media was removed and replaced with a solution of 50 μl serum-free media and 10 μl Cell Titer Blue in each well. The cell viability was evaluated after a 2 h incubation at 37 °C, 5% CO_2_ by measuring the fluorescence produced by resorufin at excitation of 550 and emission of 590, using the Synergy HT multi-detection microplate reader (Biotek, Winooski, VT, USA). The cytotoxicity of empty DOX-free PL, TfL, R8L and DualL was tested to confirm that the cytotoxicity was a result of DOX treatment (Figure S12).

### *In vivo* evaluation of therapeutic efficacy ofdual-functional liposomes

#### Subcutaneous tumor models in mice

The animal studies were performed in conformation with the NU-IACUC institutional guidelines. To study the therapeutic efficacy of Dual DOX-L *in vivo*, human ovarian carcinoma A2780 tumor model was established in nude mice. Female NU/NU nude mice (Taconic Biosciences) about four to six weeks old, were used for this purpose. An A2780 cell suspension containing 2.5 × 10^6^ cells in 100 μl sterile PBS, pH 7.4, was injected subcutaneously over the right flank of the anesthetized mice. Mice were monitored regularly for their tumor volumes and changes in body weight. Tumor volumes were measured with a vernier caliper using the following formula: Tumor Volume = [Length × (Width)^2^]/2, with length the longer axis and width the measurement perpendicular to the length (Koshkaryev et al., 2012).

#### Biodistribution studies

To evaluate the distribution of the non-modified and modified liposomes in the major organs and tumors upon administration, a pilot biodistribution study was performed. For this, subcutaneous A2780 tumors were grown in female nude NU/NU mice as described above. The tumor was considered well-established once the tumors reached an approximate volume within a range of 500-800 mm^3^. The treatment groups comprised of unmodified DOX liposome (PL DOX-L), Tf modified DOX liposome (Tf DOX-L), R8 modified DOX liposome (R8 DOX-L) and Tf and R8 conjugated Dual DOX liposome (Dual DOX-L) (four groups, *N* = 2). The mice were administered a single dose of 100 µL of the respective formulations via tail vein injection, equivalent to 10 mg/kg Doxorubicin. After a 10 h interval, the mice were anesthetized and blood was collected by cardiac puncture in heparinized tubes and spun down at 5000 RPM for 10 min, to collect the supernatant plasma. The plasma was stored at −20 °C until further analysis. Once the blood was collected, the animals were euthanized by cervical dislocation. Tumors and major organs including liver, kidney, spleen, lungs and heart were excised immediately, lightly blotted to remove excess blood and stored at −80 °C until further analysis. The tissues were later homogenized using a Qiagen tissue disruptor. A tissue homogenate solution was prepared in HBS buffer pH 7.4 (200 mg tissue/1 ml i.e. 20% w/v homogenate solution). Two hundred µl (40 mg tissue) of this homogenate solution or 50 µl of plasma were added in a 2 ml micro centrifuge tube containing the extraction buffer (100 µl of 10% v/v TritonX-100, 200 µl water and 1,500 µl of acidified isopropanol (0.75 N HCl)). DOX and its metabolites were extracted from the homogenates, overnight at −20 °C. On the following day, tubes were centrifuged at 15,000*g* for 20 min, and the supernatant was collected for analysis. About 100 µl of this supernatant was transferred directly to a 96 well plate and DOX levels in the samples were assessed using fluorescence detection at an excitation of 485 nm and an emission of 590 nm. (Li et al., 2009). DOX was quantified using a standard curve made with known concentrations of free DOX solution in the extraction buffer. Non-specific background fluorescence was corrected for using tissue samples extracted from saline-treated mice.

#### Tumor growth inhibition studies

The aim of this study was to estimate the tumor growth inhibition capability of Dual DOX liposomes in an ovarian cancer model. To achieve this, subcutaneous A2780 tumors were established in nude mice. Once the tumors grew to an approximate volume between 50 and 150 mm^3^ the animals were randomized into groups such that the starting average tumor volumes were consistent across all groups. The comparison groups used were HBS pH 7.4 (Control), free DOX, PL DOX-L, Tf DOX-L, R8 DOX-L and Dual DOX-L (six groups, *N* = 4). The mice were administered 100 µL of the respective formulations via tail vein injections equivalent to 2 mg/kg DOX in treatment groups. The doses were given every third day and the tumors were monitored for their growth and measured every third day as well. Tumor volumes were estimated as previously stated (Sawant et al., 2013). The weight of the mice was monitored throughout the study to check for signs of toxicity of DOX. The study was stopped when the control tumors reached 1000 mm^3^. Mice were euthanized at the end of the study, tumors from all groups excised and tumor weights recorded.

### Statistical analysis

The *in vitro* data were assessed for statistical significance using one-way ANOVA followed by Tukey’s *post hoc* test. All numerical data are expressed as mean ± SD, *n* = 3, from two to three different experiments. For animal study data statistical significance was assessed using One-way ANOVA and Student’s *t*-test. *p* Values were calculated with the Graph Pad Prism 5 software (GraphPad Software, Inc., San Diego, CA, USA). Numerical data are expressed as mean ± SD or mean ± SEM. *p* ≤ .05 were considered statistically significant. In figures *, **, ***, **** indicated *p* ≤ .05, .01, .001 and .0001, respectively.

## Results

### Characterization of liposomes

#### Liposome size and zeta potential

The characterization of DOX-loaded modified and unmodified liposomes in terms of their particle size and zeta potential (surface charge) is summarized (Table 1). No significant changes in mean diameter were observed across all four groups. The polydispersity (PDI) for all the liposome groups was <0.2 indicating a homogenous distribution of liposomes favoring their stability.

**Table 1.** Characterization of DOX-loaded modified and unmodified liposomes.

Sample	Mean diameter (nm)	PDI	Zeta potential (mv)
PL DOX-L	196.5 ± 1.8	0.1 ± 0.0	−31.8 ± 1.1
TfL DOXL	210.7 ± 4.4	0.1 ± 0.0	−28.0 ± 2.1
R8L DOXL	197.1 ± 1.0	0.1 ± 0.0	−7.4 ± 2.7
DualL DOXL	214.5 ± 3.3	0.1 ± 0.1	−19.2 ± 1.2

Results reported as mean ± SD.

The surface charge for PL DOX-L and Tf DOX-L was similar indicating the presence of negatively charged lipids and proteins on their surfaces. R8 DOX-L and Dual DOX-L had a more positive charge on their surface due to presence of cationic R8 peptide while Dual DOX-L were more negative than R8 DOX-L, which could be attributed to negatively charged Tf molecules on their surface.

**Figure 1.**
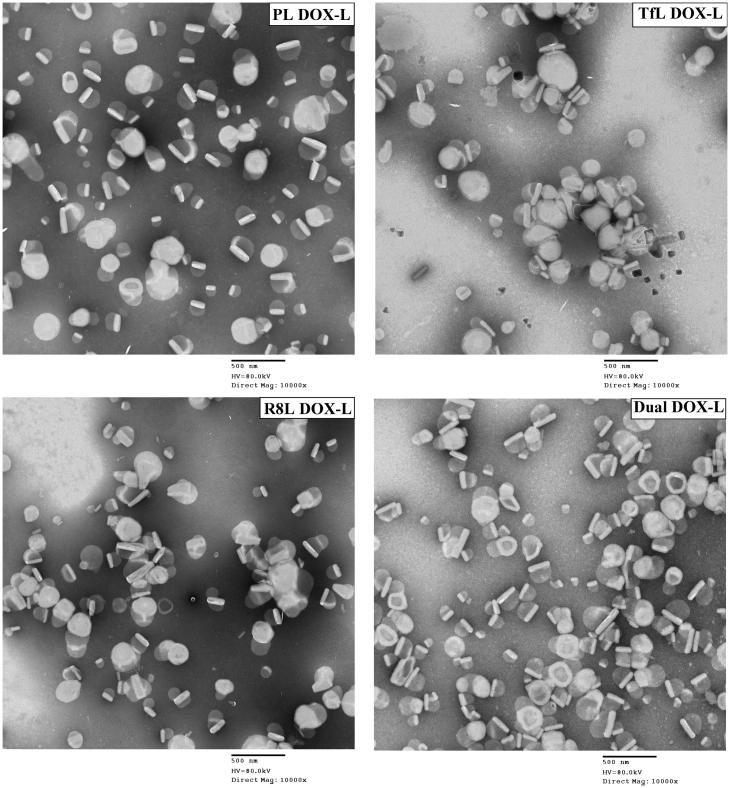
Transmission electron microscopy images of the modified and unmodified DOX-loaded liposomes. Represents 10,000× magnification, Scale bar, 500 nm.

TEM showed the spherical appearance of liposomes. Dual DOX-L had a uniform appearance similar to non-modified and single ligand modified liposomes. The DOX crystals were easily visualized trapped within the liposomes, indicating good DOX encapsulation (Figure 1).

No significant changes in size or surface charge were observed between day 1 and day 15 for all groups, indicating the liposomes were stable over a 15 day period at 4 °C (Table S4). Particle size and zeta potential of Rh-labeled Dual, single and unmodified liposomes were similar to that of DOX-loaded liposomes (Table S2).

### Measurement of percentage of DOX loaded in liposomes

DOX-liposomes (DOX-L) were prepared by an active loading technique of DOX using the ammonium sulfate gradient technique. DOX was added to the liposomes at an initial drug-to-lipid ratio of 0.16 and the loading efficiency was approximately 89.3 ± 8.7%. On an average the DOX concentration was estimated at 1.77 ± 0.2 mg/ml with 0.122 mg DOX/mg of lipid.

An *in vitro* study designed to assess DOX release from the liposomes over a 72 hour period, showed slow drug release at both pH 7.4 and pH 5.0. The release rate was slightly accelerated at pH 5 (50% within 12 h) compared to pH 7.4 (50% beyond 24 h) with a prolonged drug release at physiological pH up to 48 h (Figure S9).

### Cell association of rhodamine-labeledtransferrin-targeted dual-functional liposomes

**Figure 2.**
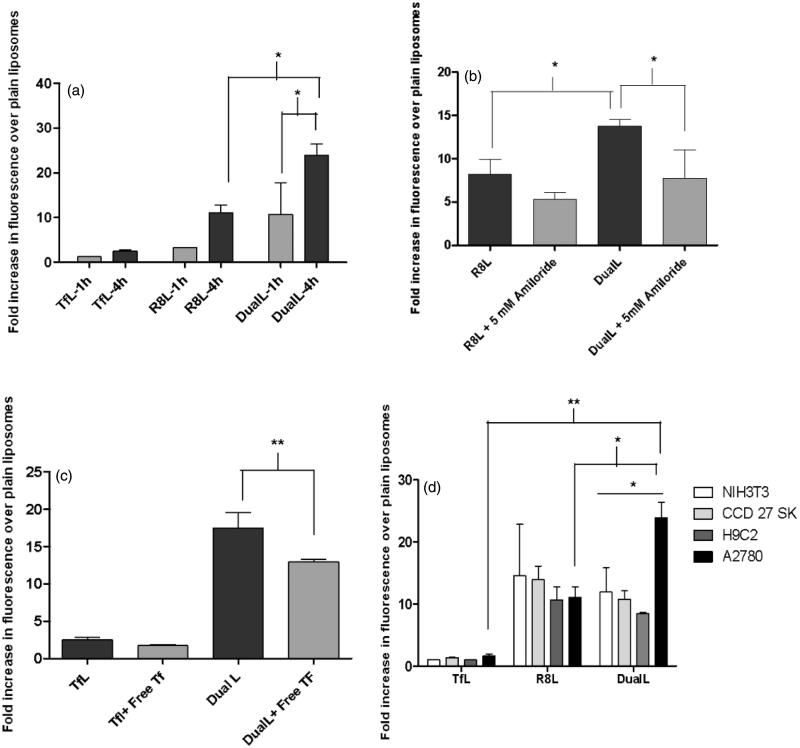
Interaction of rhodamine-labeled dual functional liposomes with A2780 cancer cells analyzed by flow cytometry. (a) Cell association of DualL with cancer cells for 1 h and 4 h treatment periods, followed by analysis by flow cytometry. (b) Effect of a macropinocytosis inhibitor, amiloride (5 mM, 30 min pre-incubation), on R8-mediated cell association for a 4 h liposome treatment period. (c) Evaluation of transferrin receptor-mediated endocytosis by competitive inhibition in presence of free excess Tf (2 mg/ml) in the treatment medium.(d) Comparison of cell association of DualL between NIH3T3, H9C2, CCD 27 SK non-cancer cells and A2780 cancer cells. Cells were incubated with liposomes at a total lipid concentration of 0.1 mg/ml. Results are plotted as fold increase in geometric mean fluorescence over plain liposomes and are mean ± SD, averaged from three separate experiments.*, **, ***, **** indicate *p* ≤ .05, .01, .001 and .0001, respectively analyzed by one-way ANOVA.

The cell association of the DualL with A2780 cancer cells and NIH3T3 cells, H9C2 cells and CCD27SK non-cancer cells was analyzed by measuring the rhodamine signal from the liposome bilayer. A time-dependent (1 and 4 h) study showed the geometric means for TfL, R8L and DualL were 2.8 ± 0.1, 7.3 ± 0.3 and 23.3 ± 9.4 after 1 h, respectively, while after 4 h the means were 4.8 ± 0.2, 28.1 ± 4.4 and 58.5 ± 12.9, respectively in A2780 cells, indicating a two-fold higher cell association was for DualL compared to R8L at 4 h exposures in cancer cells (Figure 2(a)). A time-dependent increase in the association of DualL with cancer cells was observed (*p* < .05). R8L demonstrated a nine-fold increased association and DualL showed almost a 21-fold increase, over plain liposomes. In A2780 cells, association of DualL was two-fold higher than R8L (*p* < .05), while for normal cell lines, the association of DualL was the same or even less than R8L (Figure 2(d) and Figure S5).

### Effect of macropinocytosis inhibitor on cell association of dual-functional liposomes

As it is known, that R8 internalizes by the macropinocytosis pathway, a metabolic inhibitor, amiloride, was used to examine the role of macropinocytosis in the interaction of R8-conjugated liposomes with cells. The presence of amiloride in the treatment medium reduced R8L association and significantly inhibited association of DualL when compared to their association, in the absence of amiloride (Figure 2(b)). In the absence of amiloride, the increase in association of R8L and DualL was found to be 8.2 ± 1.8- and 13.8 ± 0.7-fold, respectively. While in the presence of amiloride the fold increase was only 5.3 ± 0.8 for R8L and 7.8 ± 3.3 for DualL.

### Analysis of transferrin receptor-mediated endocytosis of dual-functional liposomes

We validated involvement of receptor mediated endocytosis (RME) in the internalization process of DualL. The specificity of receptor-mediated endocytosis of Tf-conjugated liposomes was evident from the decreased cellular association/endocytosis when cancer cells were pre-incubated with a 100-fold excess of free Tf in the medium (Figure 2(c)). The fold increase in the uptake over PL for DualL, in the absence of excess free Tf was 17.5 ± 2.1 versus 12.9 ± 0.4 in the presence of excess free Tf. This study indicated reduced association of DualL in the presence of free transferrin (*p* < .01).

### Cellular internalization of dual-functional liposomes

**Figure 3.**
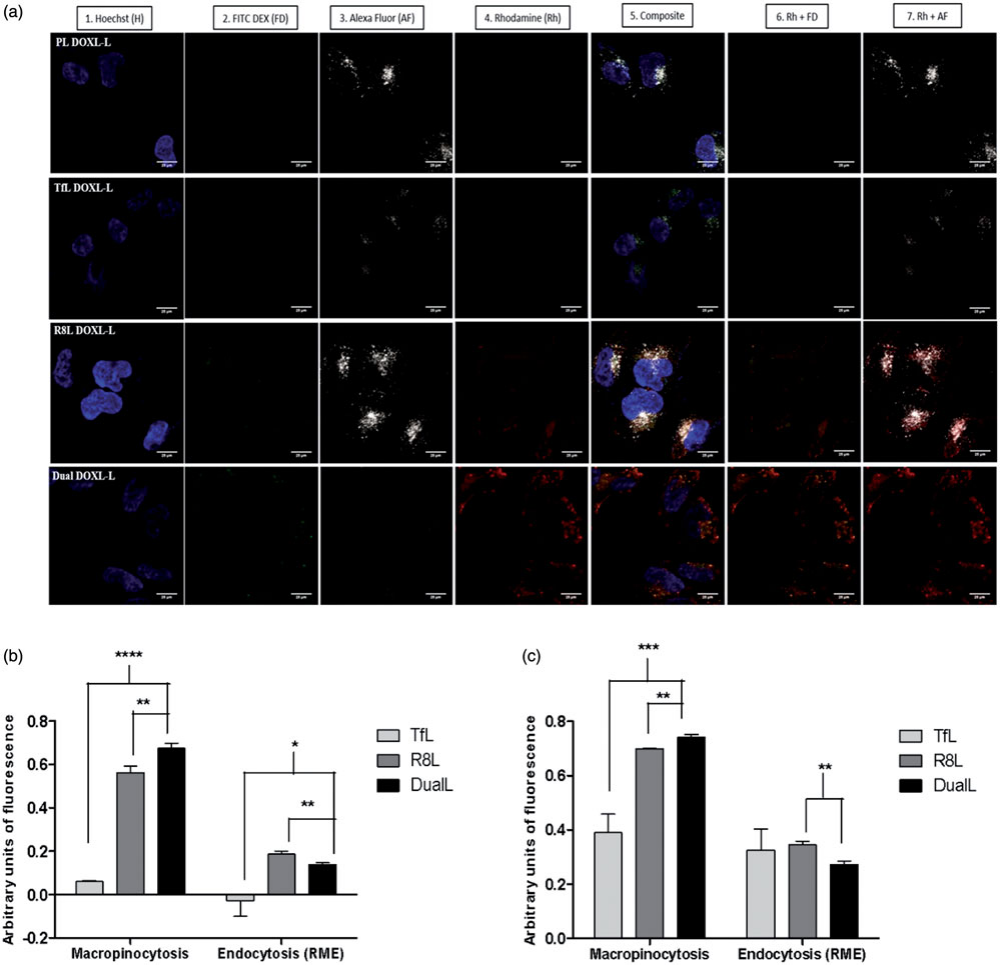
Receptor mediated endocytosis and macropinocytosis evaluation to confirm internalization of DualL. (a) A2780 cells were incubated with rhodamine-labeled PL, Tfl, R8L or DualL. Liposomes were added at a total lipid concentration of 0.1 mg/ml for a 4 h treatment period followed by analysis by confocal microscopy. (1) Nuclei stained by Hoechst 33342 at 5 µg/mL for 15 min; (2) Macropinosomes stained by FITC Dextran 70 KDa at 0.35 mg/mL for 30 min before formulation incubation; (3) Endosomes stained with Transferrin-Alexa fluor 680 at 22.5 µg/mL for 15 min; (4) Rhodamine-signal from liposomes; (5) Merged composite picture of all the fluorescence; (6) Co-localization of rhodamine with FITC Dextran (7) Co-localization of rhodamine with Alexa Fluor. Yellow signals in the merged images indicate the co-localization of the red and green, red and pinkish fluorescence represents co-localization of red and gray, respectively. Analysis of fluorescence intensity-colocalization Pearson's coefficient (b) and Mander’s coefficients (c), obtained from the merged pictures (*n* = 3) from TfL, R8L and DualL-treated cells, by Image *J* software. The results are mean ± SD averaged from three images of the same treatment. *, **, ***, **** indicate *p* ≤ .05, .01, .001 and .0001, respectively. Analyzed by Student’s *t*-test. Scale bar, 25 µm.

Confocal microscopy was used to assess the intracellular localization of the liposomes and track their mode of internalization in cancer cells. The confocal micrographs demonstrated good internalization of R8L and DualL and poor internalization of PL and TfL, inferred from the intense red rhodamine fluorescence in the cytoplasmic compartments (Figure 3(a)). Liposomes were seen localized in the cytoplasm around the nucleus and a strong red signal was observed in case of DualL-treated cells, confirming an efficient intracellular localization. For R8-conjugated R8L and DualL, a strong yellowish-orange stain in the cytoplasm (Co-localization of green macropinocytosis marker and red rhodamine signal) was observed and was significantly higher in case of DualL than R8L. PL and TfL showed poor internalization and thus poor RME. The gray Tf Alexa Fluor 680 gray stain (Figure 3(a), column 3) by itself was light in case of TfL and DualL likely due to competitive inhibition between Tf and the dye. Despite, poor internalization of the dye a pinkish stain was observed in case of DualL (Co-localization of gray and red). Complete exclusion of the blue and red stains was observed. Pearson’s and Mander’s co-localization coefficients confirmed a significantly high internalization of DualL via macropinocytosis (Figure 3(b,c)).

### Cellular internalization of DOX assessed by confocal microscopy

**Figure 4.**
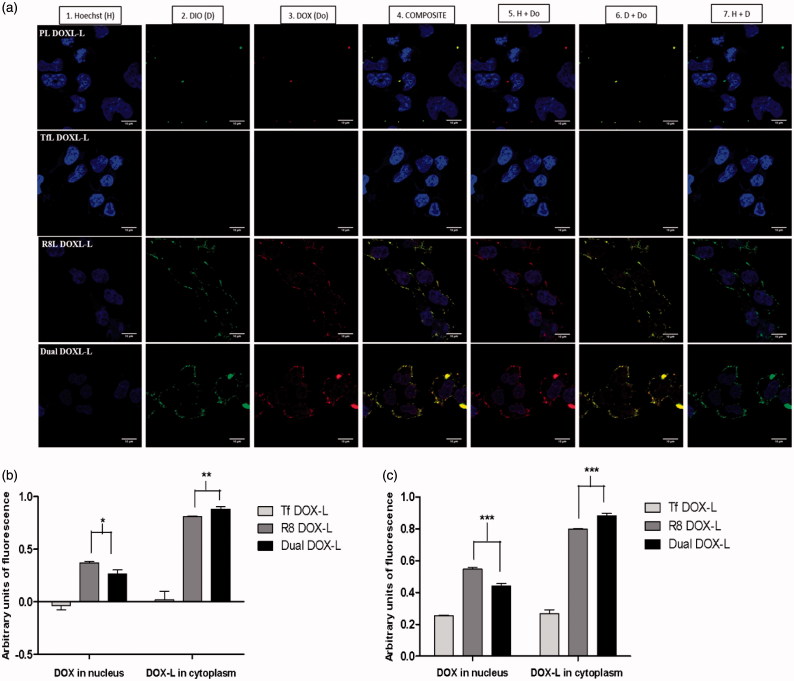
Intracellular DOX release. A2780 cells were incubated with Dio-labeled PL DOX-L, Tf DOX-L, R8 DOX-L or Dual DOX-L. Liposomes were added at a total lipid concentration of 0.1 mg/ml for 4 h treatment period followed by analysis by confocal microscopy. (a) (1) Nuclei stained by Hoechst 33342 at 5 µg/mL for 15 mins; (2) Dio stain from liposome bilayer; (3) DOX stain; (4) Merged composite picture of all fluorescence; (5) Co-localization of DOX with Hoechst; (6) Co-localization of Dio with DOX; (7) Merged image of Hoechst and Dio. Yellow signals in the merged images indicate the co-localization of the red and green indicating cytoplasmic delivery, and purple fluorescence represents co-localization of red and blue indicating nuclear delivery, respectively. Analysis of fluorescence intensity co-localization (b). Pearson's coefficient and (c). Mander’s coefficient, obtained from the merged pictures (*n* = 3) from Tf DOX-L, R8 DOX-L and Dual DOX-L-treated cells, by Image *J* software. The results are mean ± SD averaged from three images of the same treatment. *, **, ***, indicate *p* ≤ .05, .01 and .001 respectively. Analyzed by Student’s *t*-test. Scale bar, 10 µm.

We also assessed the intracellular trafficking of DOX-loaded liposomes and subsequent nuclear DOX delivery, in a separate confocal study. The confocal micrographs clearly showed good internalization of R8 DOX-L and Dual DOX-L deduced from higher green fluorescence in the cytoplasm (Figure 4(a)). Green fluorescence was seen to be most intense for Dual DOX-L, indicating enhanced cytoplasmic entry. Red fluorescence from the DOX, was strong for both R8 DOX-L and Dual DOX-L. However, a brighter and stronger red fluorescence was seen in case of the Dual DOX-L. Strong purple staining in the nucleus (overlap of red DOX with blue nuclear stain Hoechst) for R8 DOX-L and Dual DOX-L indicated good nuclear delivery of DOX. Although, the purple stain was slightly stronger in case of R8 DOX-L than Dual DOX-L, an intense yellow stain (from overlap of green and red) in the cytoplasm was observed, for Dual DOX-L. Complete exclusion blue and green stains confirmed localization of liposomes in the cytoplasm and entry of DOX alone in the nucleus which is its site of action (Figure S10). Pearson’s and Mander’s co-localization coefficients indicated high nuclear DOX delivery for R8 DOX-L, but better cytoplasmic accumulation in the perinuclear compartment of Dual DOX-L (Figure 4(b,c)).

### *In vitro* cytotoxicity of DOX-loaded liposomes using cell-titer blue assay

**Figure 5.**
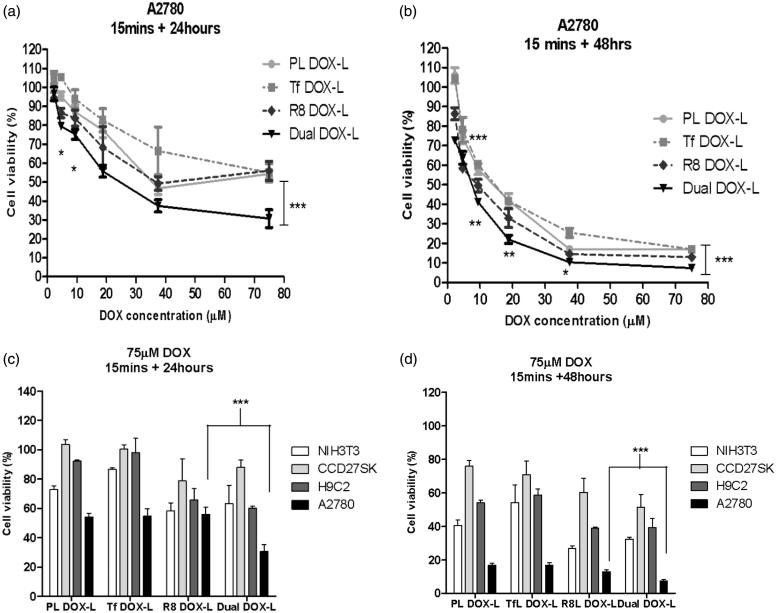
Effect of dual functional DOX-loaded liposomes on cell death in cancer and non-cancer cells. Assessment of cell viability of A2780 cells treated with DOX-loaded PL DOX-L, Tf DOX-L, R8 DOX-L and Dual DOX-L, at DOX concentration of 0.2—75 µM for 15 min followed by 24 (a) or 48 h (b) incubations. Comparison of cell death in A2780 cancer and NIH3T3, H9C2, CCD27Sk non-cancer cells at 75 µM. Cells treated with DOX-loaded PL DOX-L, Tf DOX-L, R8 DOX-L and Dual DOX-L for 15 min followed by 24 (c) or 48 h (d) incubations. Results obtained as mean ± S.D. from three separate experiments. * indicates *p* < .05, ** indicates *p* < .01, *** indicates *p* < .001analyzed by one-way ANOVA.

Once it was established that DualL showed high association, good internalization and better selectivity towards cancer cells compared to non-cancer cells, the next goal was to determine their ability to enhance the cytotoxicity of DOX. Here, PL DOX-L, Tf DOX-L, R8 DOX-L and Dual DOX-L were tested for their effect on cell viability. Dual DOX-L treatment produced higher cytotoxicity than all the other treatment groups in A2780 cancer cells. A significant increase in the cytotoxic effects of Dual DOX-L in comparison to R8L, was observed after both 24 or 48 hours incubation (*p* < .001 at the highest tested dose). Dual DOX-L showed approximately 30% and 10% more cell death than R8 DOX-L after 24 or 48 h, respectively at the highest tested dose of DOX. Dual DOX-L demonstrated 30.6 ± 4.7% and 7.3 ± 1.1% cell viability compared to 55.9 ± 5.0% and 12.9 ± 1.2% for R8 DOX-L, at the highest tested DOX dose of 75 µM at 24 and 48 h, respectively (Figure 5(a,b)). In non-cancer cells, Dual DOX-L at DOX dose of 75 µM, demonstrated 63.2 ± 12.5% and 32.2 ± 1.4% cell viability in NIH3T3 cells; 60.2 ± 1.1% and 39.3 ± 5.2% in H9C2 cells; 87.9 ± 5.0% and 51.2 ± 7.7% in CCCD27SK cells, at 24 and 48 h, respectively (Figure S11). No significant difference between cytotoxicity of Dual DOX-L and R8 DOX-L was as observed in normal cells, in contrast to the observation in A2780 ovarian cancer cells (Figure 5(c,d)). DOX-free empty formulations showed that PL and TfL empty liposomes were nontoxic to the cells. In cells treated with R8L and DualL, the highest toxicity seen in both cancer as well as non-cancer cells, was approximately 20% cell death after 24 h post-treatment incubation and 25–30% after 48 h incubation (Figure S12). The 20–30% cell death observed with these empty liposomes was insignificant as compared to the cytotoxic effects of R8 DOX-L and Dual DOX-L.

### Biodistribution of DOX in tumors and major organs

**Figure 6.**
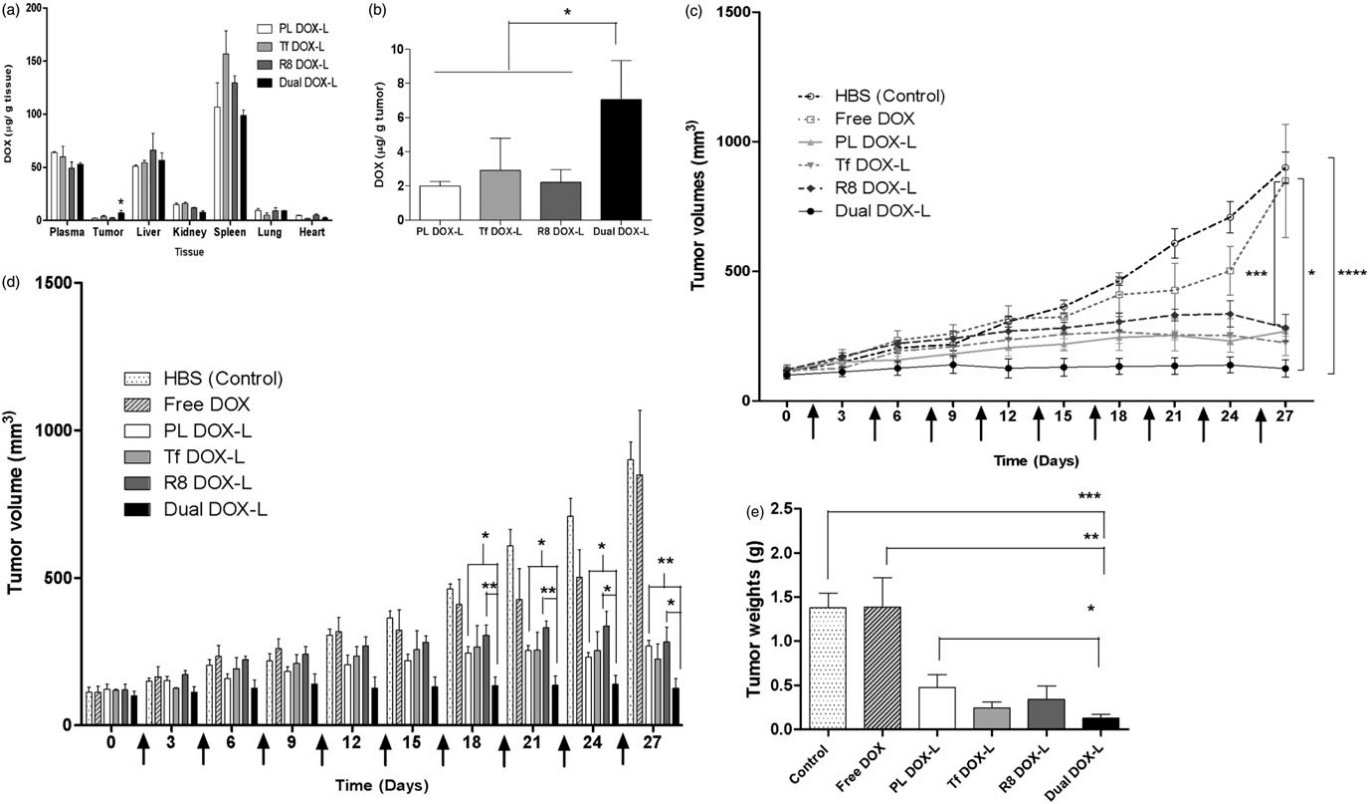
Evaluation of *in vivo* therapeutic efficacy of Dual DOX-L. Biodistribution of Dox in mice bearing A2780 tumors. (a) Represents distribution of DOX in major organs. (b) Represents distribution of DOX in tumors across 4 tested groups. A single 10 mg/kg i.v. tail injection of PL DOX-L, Tf DOX-L, R8 DOX-L and Dual DOX-L was administered. After 10 h the mice were sacrificed and major organs and tumors were collected. *N* = 2 animals per group ± SD. Analyzed by one-way ANOVA where *p < 0.05. Effect of i.v. administration of liposomes on tumor growth in nude mice bearing A2780 tumors. Treatment groups were HBS (control), Free DOX, PL DOX-L, Tf DOX-L, R8 DOX-L, and Dual DOX-L. Arrows indicate day of treatment with the formulations given after tumors reached 50–150 mm^3^. (*n* = 4, mean ± SEM). Calculated from day 0 to day 27 of treatment. (c) Represents a line graph showing the trend of tumor growth. (d) Represents a column graph of tumor volumes for all groups. (e) A2780 tumor weights at the end of the study. Treatment was stopped and tumors were excised when the average tumor volume in the control group reached 1000 mm^3^. Represented as mean ± SEM. *, **, ***, **** indicate *p* ≤ .05, .01, .001 and .0001, respectively analyzed by Student’s *t*-test.

A pilot biodistribution study was performed to investigate the distribution of DOX via DOX-loaded modified and unmodified liposomes *in vivo*. Plasma, tumor and the major organs from saline-injected mice were used as controls to eliminate the background fluorescence. Ten hours post-injection, all four DOX-loaded liposome groups were circulating in the blood estimated from the DOX extracted from plasma. DOX accumulated at significantly higher levels in liver and spleen compared to other major organs or tumors for all treatment groups. Levels of DOX distribution in lungs and heart were negligible for all groups. Dual DOX-L liposomes accumulated in significantly higher quantities in the tumors compared to the other treatment groups (Figure 6(a,b)). 2% of the injected dose accumulated in the tumors in case of Dual DOX-L treatment versus <1% for other formulations (Figure S13).

### Tumor growth inhibition study

The therapeutic efficacy of Dual DOX-L *in vivo* was estimated in a tumor growth inhibition experiment. At the tested dose, all liposomal DOX groups showed an inhibitory effect on tumor growth compared to the control tumors and free DOX treated tumors. Dual DOX-L was most effective in controlling the tumor growth throughout the 27-day study (as early as the second dose). All four DOX liposome groups showed similar tumor growth trends initially and changed towards the middle of the study (Figure 6(c,d)). From day 18 onwards, a significant difference in tumor volumes developed between Dual DOX-L, PL DOX-L and R8 DOX-L. At the end of the study the tumor volumes were 900.3 ± 60.0 mm^3^ for control tumors, 848.6 ± 218.2 mm^3^ for Free DOX, 268.4 ± 19.2 mm^3^ for PL DOX-L, 224.8 ± 50.3 mm^3^ for Tf DOX-L, 281.6 ± 51.6 mm^3^ for R8 DOX-L and 124.9 ± 33.69 mm^3^ for Dual DOX-L (Figure 6(c,d)). Dual DOX-L treatment was consistently more effective in controlling tumor growth than treatments throughout the study (Figure S14). At the end of the study, the mice were sacrificed and tumors were isolated. The tumor weights for all liposomal treatments were significantly lower than control and free DOX treatment groups (Figure 6(e)). Dual DOX-L treated tumor tissues were significantly smaller compared to control, free DOX and PL-DOX-L-treated tumors. The mouse weights recorded throughout the study, were fairly constant indicating no apparent toxicity of the formulations (Figure S15).

## Discussion

The overall goal of this project was aimed at the development of a drug delivery platform with improved intracellular delivery and enhanced cancer cell-specificity. Our earlier studies showed that surface-modification of DOXIL^®^ with the AR-CPP, R8, helped the liposomes to escape endosomal capture and enhanced therapeutic efficacy, by achieving better nuclear DOX delivery (Biswas et al., 2013). The strong cationic nature of R8 combined with its nonspecificity for cancer cells however, could lead to accumulation of the liposomes in non-target tissues, defeating the purpose of intracellular drug delivery in cancer cells. To address this challenge, we designed DualL, by adding a second ligand, Tf, selective for TfRs, known to be over-expressed on the cell-surfaces of A2780 ovarian cancer cells (Koshkaryev et al., 2012; Sawant et al., 2013).

To validate the concept of dual-functional ligands on the liposome-surface, rhodamine-labeled liposomes (same lipid composition as DOXIL^®^) surface-modified with varying densities of Tf and R8 were tested. DualL with the 2 mol% R8-PEG_2K_-PE and 0.5 mol % Tf-PEG_3.4 K_-PE showed the best association with cancer cells, in a time-dependent manner (Figures S2 and S3 and Figure 2(a)). DualL exhibited significantly higher association with cancer cells than R8L and this association was inhibited in the presence of amiloride, indicating the contribution of R8 in the internalization of DualL (Figure 2(a,b)). When free excess Tf was added to the medium, uptake of DualL was inhibited validating competitive inhibition due to Tf activity and supporting the role of Tf in the uptake of DualL (Figure 2(c)). R8L showed a similar association pattern with both, cancer and non-cancer cells, but DualL had a significantly higher association only with A2780 cancer cells (Figure 2(d) and Figure S5). Confocal microscopy confirmed the internalization of DualL in cancer cells by both, RME and macropinocytosis. DualL showed significantly higher accumulation in the cytoplasm compared to TfL and R8L (Figure 3(a–c) and Figure S6a–c). These studies confirmed better internalization of DualL in cancer cells by contribution from both functional ligands.

Based on these findings, we explored the therapeutic efficacy of DOX-loaded DualL. DOX was actively loaded in Rh-free liposomes using an ammonium-sulfate gradient technique to achieve stable liposomes and mimic DOXIL^®^, followed by surface modification with R8 and Tf. Dual DOX-L exhibited consistent particle size, surface morphology and surface charge (Table 1 and Figure 1). A greater negative charge on their surface, may help to minimize their nonspecific interactions *in vitro* and *in vivo* compared to the less negatively charged R8 DOX-L. Results from confocal microscopy for Dual DOX-L treatment, supported good nuclear delivery of DOX, comparable to that by R8 DOX-L possibly by favoring endosomal escape. Greater accumulation of Dual DOX-L in the cytoplasm, compared to R8 DOX-L and Tf DOX-L was evidence of good internalization of these liposomes in cancer cells (Figure 4(a–c)). A challenge that was addressed next, was to establish the link between good internalization of Dual DOX-L, good nuclear DOX delivery and their subsequent cytotoxic effects towards cancer cells. A cell viability assay performed for this purpose showed that at highest tested concentration of DOX (75 µM), Dual DOX-L liposomes showed significant cytotoxicity in cancer cells compared to all other treatment groups, with just 15 min of incubation with cells. The cytotoxic effect was amplified after 48 h in cancer cells (Figure 5(a,b)) while, in non-cancer cells tested under the same conditions, no differences in cytotoxicity among the treatment groups, was observed (Figure 5(c,d) and Figure S11). These results confirmed, Tf targeting promoted better cytotoxicity towards A2780 cancer cells, *in vitro,* when combined with the intracellular delivery effect of R8.

In the final step of our study, the therapeutic efficacy of Dual DOX-L was tested *in vivo* in an A2780 ovarian tumor xenograft model. The tumor growth inhibition study was initiated at an early stage of tumor growth, to test the therapeutic potential of the liposomes. With the help of literature survey a 2 mg/kg DOX dose was chosen (Gao et al., 2005; Biswas et al., 2013; Zhao et al., 2013; Apte et al., 2014; Lin et al., 2014; Sriraman et al., 2016; Zahmatkeshan et al., 2016). Compared to the control and free DOX-treated tumors, all the liposomal DOX groups showed effective control of tumor growth *in vivo*. Dual DOX-L treatment was most effective in controlling the tumor growth throughout the study, from the second injection onwards, by allowing almost no increase in tumor size. Towards the end of the treatments, significant differences were seen between the tumor sizes of Dual DOX-L treated group and PL DOX-L and R8 DOX-L (Figure 6(c,d)). Dual DOX-L were better than Tf DOX-L in controlling tumor size, although no significant differences could be established, possibly attributable to the potency of DOX and the effectiveness of the overall treatment of DOX-L itself. However, as the Dual DOX-L treatment was significantly better than PL DOX-L and R8 DOX-L treatments and Tf DOX-L treatment did not show any significant differences in tumor sizes, Dual DOX-L could be considered a more efficient treatment than Tf DOX-L. At the end of the study, Dual DOX-L treated tumors weighed the least compared to all other groups, with tumor weights significantly lower than the control (HBS), Free DOX and PL-DOX-L treatment groups (Figure 6(e)). Low tumor weights for all liposomal treatment groups compared to control (HBS) and Free DOX, confirmed the effectiveness of the DOX-L treatment. The pilot biodistribution study showed enhanced accumulation of Dual DOX-L in tumor tissues at a 10 mg/kg DOX dose after a 10 h post-treatment interval (Figure 6(a,b)). The largest fraction of the drug was detected either circulating in the plasma or in the liver, followed by spleen and negligible amounts in the lungs and heart (Figure 6(a)). The % injected dose of DOX accumulating in tissues, was highest for dual-DOX-L treatments in the tumor (Figure S13), indicating advantage of the Tf R8 combination on accumulation in tumors. All results represent a clear translation of *in vitro* effects into an enhanced *in vivo* therapeutic potential of the dual-targeted liposomes which is in agreement with data published recently by Liu et al. illustrating a promising transferrin–cell penetrating peptide–sterically stabilized liposome (TF-CPP-SSL) system. This study exploited properties of PEG by optimizing its chain length and molecular weight to make the dual-targeted liposomes more efficient to deliver DOX for glioma therapy. The authors concluded that using a longer PEG chain with Tf could mask nonspecificity of the CPP during circulation, while at target site, the flexible PEG chains could bring CPP closer to the cell membrane promoting electrostatic interactions translating into an advanced dual-targeted delivery system for glioma (Liu et al., 2017). Overall, our studies collectively suggest that Dual DOX-L was most effective to target and tackle A2780 ovarian cancer *in vitro* and *in vivo*, and is a step closer to building a good, efficient drug delivery system.

## Conclusions

Here, we aimed to develop a therapeutic platform that enhanced the positive attributes of an existing, moderately effective system, by minimizing its limitations. Dual DOX-L system endowed with passive targeting (by virtue of size via EPR effect), better tumor penetration and intracellular delivery (by R8) and active targeting of TfRs over-expressed on the cancer cell-surface (by Tf via RME of TfRs) worked effectively to enhance therapeutic potential of DOX. The studies designed to investigate the role of each of the above components, clearly justified their importance. Irrespective of the cell-type, R8 showed improved intra-cellular delivery of the therapeutic cargo, consistent with a wide variety of research published on AR-CPPs and their application in the liposome drug delivery field (El-Sayed et al., 2009; Schmidt et al., 2010; Futaki et al., 2013). TfR targeting using Tf, has also emerged as a popular cancer-cell targeting approach based on recent published literature (Kolhatkar et al., 2011; Mehra et al., 2013; Tortorella & Karagiannis, 2014; Nogueira et al., 2015), also corroborated by results from our study. Dual DOX-L showed promise for effectiveness in ovarian cancer both *in vitro* and *in vivo* and our encouraging results further validate the effectiveness of the dual targeted system.

## Supplementary Material

IDRD_Torchilin_et_al_Supplemental_Content.docx
