# Supplementary material for: Transferrin and octaarginine modified dual-functional liposomes with improved cancer cell targeting and enhanced intracellular delivery for the treatment of ovarian cancer
Source: Drug Deliv. 2018 Feb 12;25(1):517–32. doi: 10.1080/10717544.2018.1435747 (PMC6058534; doi:10.1080/10717544.2018.1435747)
Supplement: IDRD_Torchilin_et_al_Supplemental_Content.docx [file IDRD_A_1435747_SM8989.docx]

**Transferrin and octaarginine modified dual-functional liposomes with improved cancer cell targeting and enhanced intracellular delivery for the treatment of ovarian cancer**

**Authors:** ^1^Pranali Deshpande, ^1^Aditi Jhaveri, ^1^Bhushan Pattni, ^2^Swati Biswas and ^1^Vladimir Torchilin

^1^Center for Pharmaceutical Biotechnology and Nanomedicine, Northeastern University, Boston, MA 02115, USA.

^2^Department of Pharmacy, Birla Institute of Technology & Science-Pilani , Hyderabad Campus, Jawahar Nagar, Shameerpet, Hyderabad 500078, Telangana, India.

Corresponding Author address: Dr. Vladimir P. Torchilin,

Center for Pharmaceutical Biotechnology and Nanomedicine, Northeastern University, 360 Huntington Ave, 140 The Fenway, Room 216, Boston, MA 02115, USA.

E-mail: v.torchilin@neu.edu; Tel: 617-373-3206; Fax 617-373-7509

### Supplementary material

## Characterization of Rhodamine-PE labelled-liposomes

**Liposome size and zeta potential**

Liposome size and size distribution was determined by the dynamic light scattering using a Coulter N4 MD submicron particle analyzer (Beckman Coulter, Inc., Fullerton, CA). Zeta potential of the liposomal preparations were determined using 90 PLUS particle size analyzer with ZETA PALS System, Brookhaven Corporation (Holtsville, NY) at 25^0^C. **(Supplementary Table.1)**

## Effect of ligand (Tf) density on interaction of liposomes with cancer cells by FACS

The optimum density of the targeting ligand needed to bring out the maximal targeting effect of the liposomes towards A2780 cancer cells was analyzed first. To test the dual functionality hypothesis, Tf-modified liposomes with varying ligand densities of 0.05 mol%, 0.1 mol%, 0.25 mol%, 0.5 mol% and 1 mol% Tf were conjugated to rhodamine-labelled non-PEGylated liposomes by the post-insertion technique. The cell association of these liposomes was assessed by flow cytometry analysis. A2780 cells were allowed to grow until 80% confluence in a T75 flask and after first few passages, 0.3X10^6^- 0.5X10^6^ cells per well were seeded in 12 well-plates. After overnight incubation, the cells were treated with liposomes grafted with different mol% of Tf, at a dose of 0.1 mg/ml of total lipids in serum-free medium and incubated for 4 hours. The cells were washed with PBS pH 7.4 two-three times followed by trypsinization. The cell pellet was ultimately re-suspended in PBS pH 7.4, before reading the samples for rhodamine fluorescence using a BD FACS Caliber flow cytometer. The red rhodamine fluorescence was recorded in the FL-2 channel. The cells were gated using forward (FSC-H)-versus side-scatter (SSC-H) to exclude debris and dead cells before analysis of 10,000 cell counts. Data analysis performed using Cell Quest software (Becton, Dickinson and Company, Franklin Lakes, NJ) **(Supplementary Figure S1)**.

## Effect of varying densities of R8 on cell interaction of Tf-targeted liposomes

To obtain the optimum activity of both functional ligands Tf and R8, on the liposome surface, dual ligand functional liposomes (DualL) were initially tested with varying ligand densities of R8 in combination with fixed amount of Tf (from previous experiment). The peptide density range used was 0.5 mol %, 1 mol%, 2mol%, 3mol% and 4 mol% of R8-PEG_2k_-PE conjugated to liposomes by post-insertion technique. The Tf– PEG_3400_–PE co-polymer was grafted onto these liposomes at a fixed concentration of 0.5 mol% Tf. A2780 cells were seeded in 12 well-plates, about 0.3X10^6^- 0.5X10^6^ cells per well after first few passages. The cells were treated with the above mentioned single or dual functional treatment groups at 0.1 mg/ml total lipid/ ml of serum-free medium. The cellular association of the combination liposomes was tested using flow cytometry using same protocol mentioned in the previous section **(Supplementary Figure S2)**.

## Effect of PEGylation on uptake of Tf-conjugated single ligand liposomes

The influence of PEGylation on the association of single ligand modified TfLs, with cancer cells was also analyzed. Here, the competitive inhibition of uptake of TfLs was also assessed in the presence of excess Tf in the medium. Holo-Tf was added in serum-free media at a concentration of 2 mg/mL before treatment with liposomes. 0.3X 10^6^- 0.5X10^6^ A2780 cells were seeded per well in 12 well plates. After overnight incubation, the cells were incubated with or without free Tf for about 15 minutes, before treatment with PL, Non-PEGylated PL, TfL and Non-PEGylated TfL at 0.1 mg/ml of total lipids in per ml of medium for a 4 hour incubation period. The excess Tf was incubated with the cells throughout the experiment. At the end of the incubation period, the media was removed and the cells were washed two to three times with ice-cold PBS (pH 7.4) to remove the free formulation. The cells were then detached using trypsin, followed by deactivation with serum. The cells were washed again with PBS and centrifuged at 1000rpm for 5 minutes. The cell pellet was re-suspended in PBS pH 7.4 before reading the samples for rhodamine fluorescence using a BD FACS Caliber flow cytometer as mentioned above **(Supplementary Figure S3)**.

## Association of liposomes with normal or non-cancer cells

The cell association of single ligand and dual ligand modified liposomes with non-cancer cells that do not over-express Tf receptors was analyzed by flow cytometry analysis to establish targeting effects towards cancer cells. The involvement of the macropinocytosis pathway in the association and internalization of dual liposomes in non-cancer cells was assessed by incubating the cells with amiloride (5mM) for 30 minutes prior to the addition of the formulation. NIH 3T3, H9C2 and CCD 27 SK cells that do not over-express TfRs, were allowed to grow until 80% confluence in a T75 flask. After first few passages, 0.3X 10^6^ to 0.5X10^6^ cells per well were seeded in 12 well-plates. After overnight incubation, the cells were treated with PL, TfL, R8L or DualL at a dose of 0.1 mg/ml of total lipids in per ml of serum-free medium with or without amiloride for a 4 hour incubation period. 5mM amiloride was incubated with the cells throughout the experiment. The media was removed after the incubation period was completed and the cells were washed two to three times with ice-cold PBS (pH 7.4) to remove the formulation. The cells were then detached using trypsin, followed by deactivation with serum. The cells were washed again with PBS and centrifuged at 1000rpm for 5 minutes. The cell pellet was re-suspended in PBS pH 7.4 before reading the samples for rhodamine fluorescence using a BD FACS Caliber flow cytometer as mentioned above **(Supplementary Figure S4)**.

## In vitro drug release

To estimate the release rate of encapsulated DOX from the liposomes, a drug release study was performed using a dialysis technique. Briefly, 250µl of free Doxorubicin and DOX-loaded liposomes, equivalent to 500µg DOX, were placed in a dialysis bag (Mw cut-off of 10,000 Da). For DOX-loaded liposomes the un-encapsulated drug was removed prior to the drug release testing. The dialysis bags were sealed at both ends and dialyzed against PBS with 10% fetal bovine serum at two different pHs of 7.4 and 5 under continuous stirring at 100 rpm at 37°C. At the predetermined time intervals of 0, 0.25, 0.5,1, 2, 4, 6, 8, 12, 24, 48 and 72 hours, 1 ml aliquots were withdrawn from the dialysis buffer and replaced with equal volume of fresh medium. The DOX concentrations were calculated based on the fluorescence intensity of DOX at excitation of 485 nm and emission of 590 nm. The cumulative amount of DOX released over 72 hours was quantified, and results were plotted against time **(Supplementary Figure S7)**.

## Cytotoxicity of empty liposomes

To confirm that the toxicity of DOX-loaded liposomes towards the cells was caused by the encapsulated DOX and not the carrier itself, empty liposomes without Doxorubicin were tested on cancer and non-cancer cells. A 6 to 400 μg/ml lipid concentration range of empty PL, TfL, R8L and DualL was analyzed. The formulations were incubated with the cells for 4 hours in serum-free media followed by removing the formulation and further incubating the cells in complete media for 24 and 48 hours. After 24 or 48 h incubations, the media was removed and replaced with a solution of 50 μl serum-free media and 10 μl Cell Titer Blue in each well. The cell viability was evaluated after 2 h of incubation at 37^0^C, 5% CO_2_ by measuring the fluorescence produced by resorufin at excitation of 550 and emission of 590, using the Synergy HT multi-detection microplate reader (Biotek, Winooski, VT) **(Supplementary Figure S9)**.


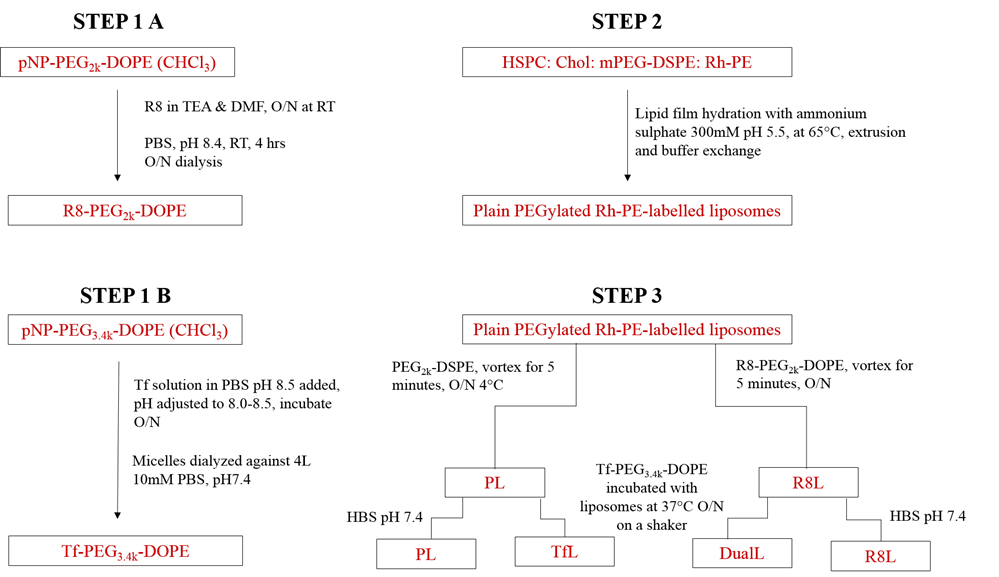


**Supplementary Figure S1.** Schematic representation of synthesis of single and dual ligand modified rhodamine-labelled liposomes

| Liposomes (Abbreviation) | Composition of liposomes (mol %) | | | | PEG_2000_-DSPE  (mol %) | R8-PEG_2000_-PE  (mol %) | Tf-PEG_3400_-PE (mol %) |
| --- | --- | --- | --- | --- | --- | --- | --- |
|  | HSPC | Chol | mPEG-DSPE | Rh-PE |  |  |  |
| Non-PEG-PL | 61.48 | 38.21 | - | 1 | - | - | - |
| PL | 59 | 38.21 | 2 | 1 | 2 | - | - |
| R8L | 59 | 38.21 | 2 | 1 | - | 2 | - |
| TfL | 59 | 38.21 | 2 | 1 | 2 | - | 0.5 |
| DualL | 59 | 38.21 | 2 | 1 | - | 2 | 0.5 |

**Supplementary Table 1.** Lipid composition of rhodamine-labelled liposomes

| **Liposomes** | **Mean diameter ± SD (nm)** | **Zeta Potential ± SD (mV)** |
| --- | --- | --- |
| Plain Liposomes | 206.8 ± 58.8 | -46.1 ± 0.9 |
| Tf Liposomes | 211.2 ± 74.5 | -49.5 ± 0.1 |
| R8 Liposomes | 211.6 ± 34.7 | 6.2 ± 2.9 |
| Dual Liposomes | 212.1 ± 30.6 | 1.3 ± 1.1 |

Supplementary Table 2. Size distribution and zeta potential analysis of PL, TfL, R8L & DualL


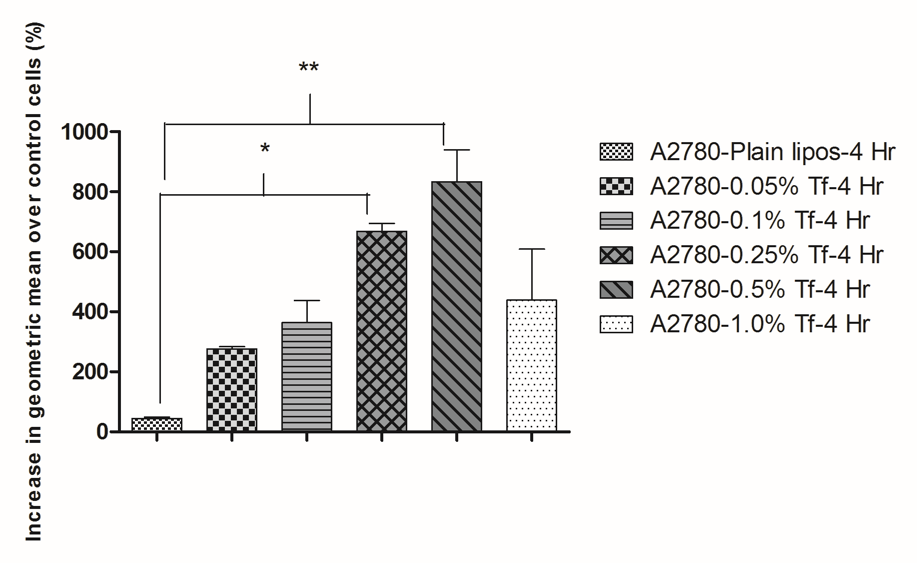


**Supplementary Figure S2.** Effect of density of Tf on liposomes surface on interaction with cancer cells.

A2780 cells were incubated with rhodamine-labelled liposomes bearing various ligand densities of Tf on their cell surface. The liposomes were added at a total lipid concentration of 0.1 mg/ml for 4 hours in serum-free media followed by analysis by flow cytometry. The results are plotted as increase in geometric mean fluorescence over control cells (%) and are mean ± SD, averaged from three separate experiments. * indicates *p*<0.05, ** indicates *p*<0.01, analyzed by One-way ANOVA.

**
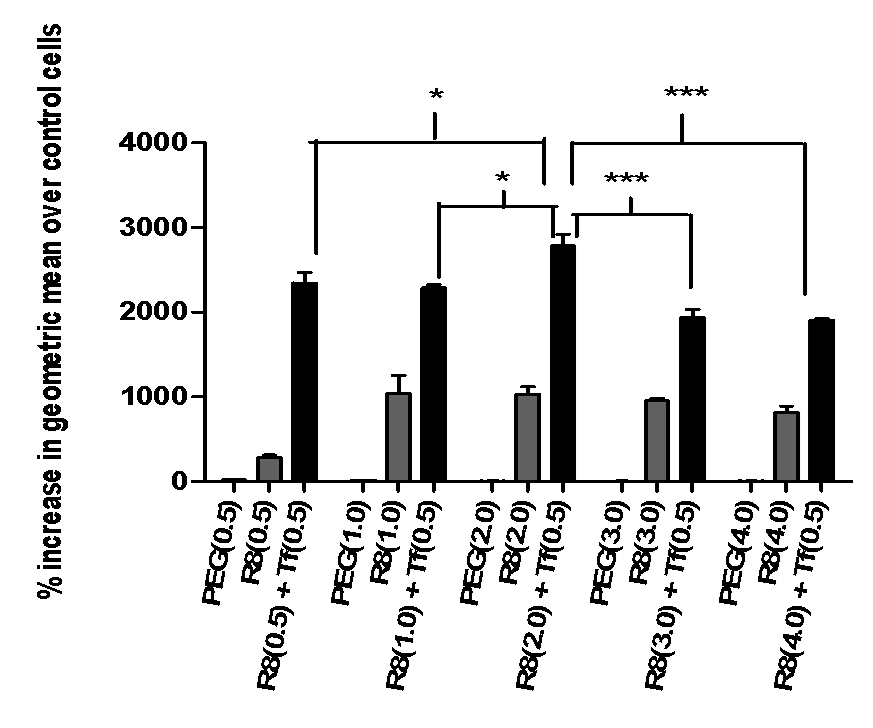
**

**Supplementary Figure S3.** Effect of density of R8 on Tf-coupled liposomes association with cells

A2780 cells were incubated with rhodamine-labelled transferrin (0.5 mol %) liposomes bearing various ligand densities of R8 on their liposome-surface. The liposomes were added at a total lipid concentration of 0.1 mg/ml for 4 hours in serum-free media followed by analysis by flow cytometry to obtain the optimum density of R8 required to make dual functional DualL. The results are plotted as increase in geometric mean fluorescence over control cells (%) and are mean ± SD, averaged from separate experiments. * indicates *p*<0.05, *** indicates *p*<0.001, analyzed by One-way ANOVA.


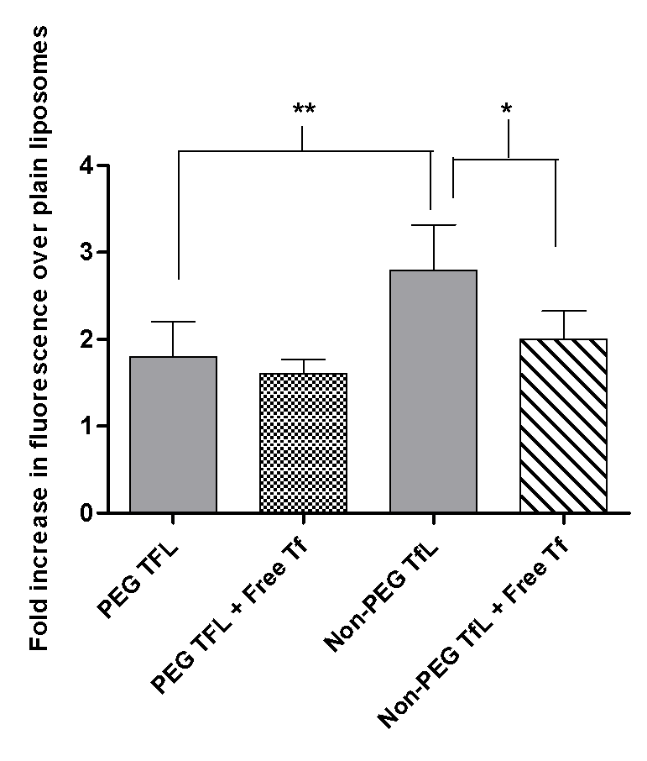


Supplementary Figure S4. Effect of PEGylation on association of Tf-conjugated liposomes.

A2780 cells were incubated with rhodamine-labelled PEGylated TfL and non-PEGylated TfL in the presence or absence of free transferrin. Cells were pre-incubated with free Tf for 15 mins before liposomes were added at a total lipid concentration of 0.1 mg/ml for 4 h treatment period followed by analysis by flow cytometry. The results are plotted as fold increase in geometric mean fluorescence plain liposomes and are mean ± SD, averaged from three separate experiments. * indicates *p*<0.05, ** indicates *p*<0.01, analyzed by One-way ANOVA.


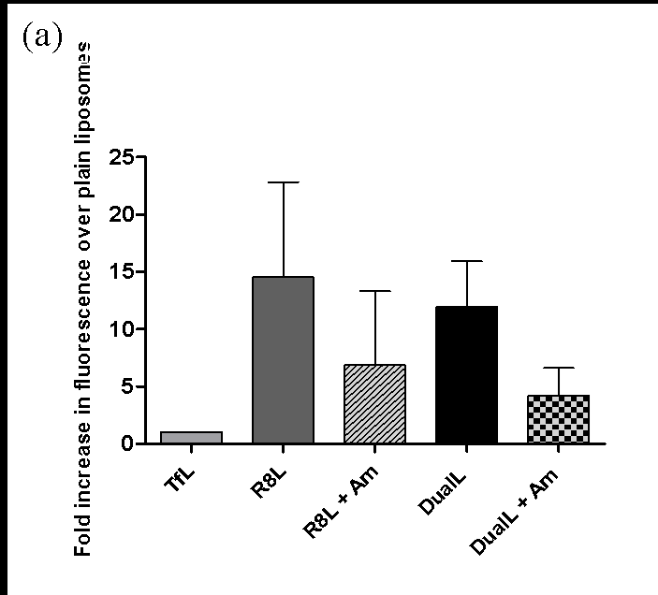

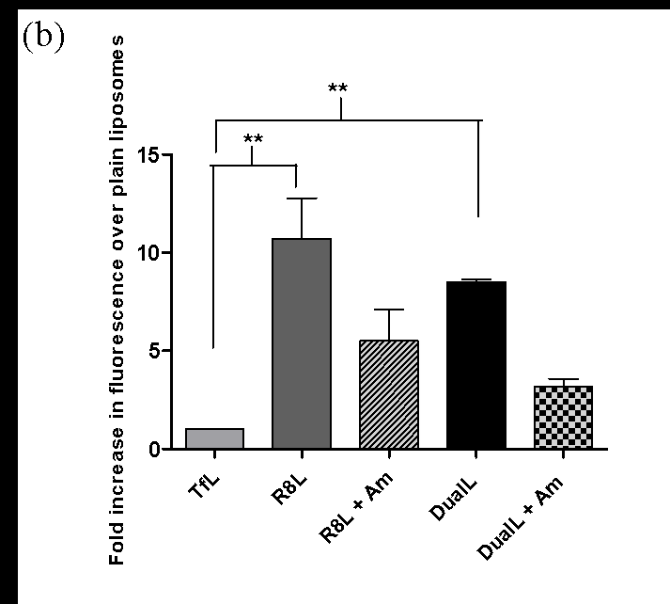


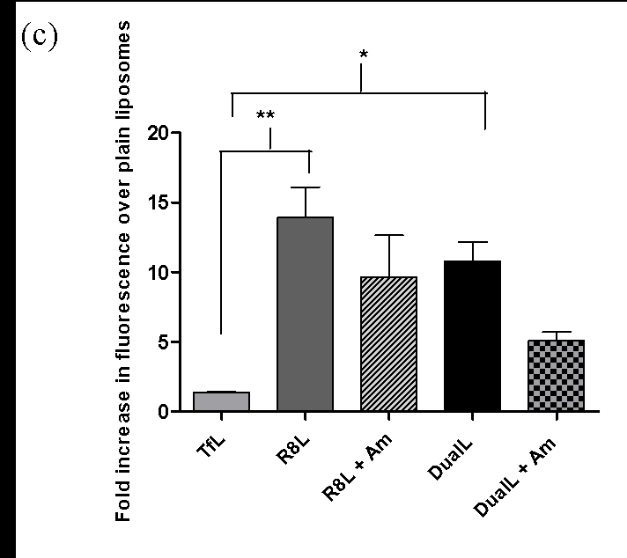


Supplementary Figure S5. Evaluation of association of single and dual functional liposomes with non-cancer cells.

Liposomes were added at a total lipid concentration of 0.1 mg/ml for a 4 h treatment period followed by flow cytometry of (A) NIH3T3 cells; (B) H9C2 cells; (C) CCD 27 SK cells; These results are mean ± SD averaged from three experiments. * indicates *p*<0.05, ** indicates *p*<0.01, analyzed by One-way ANOVA.


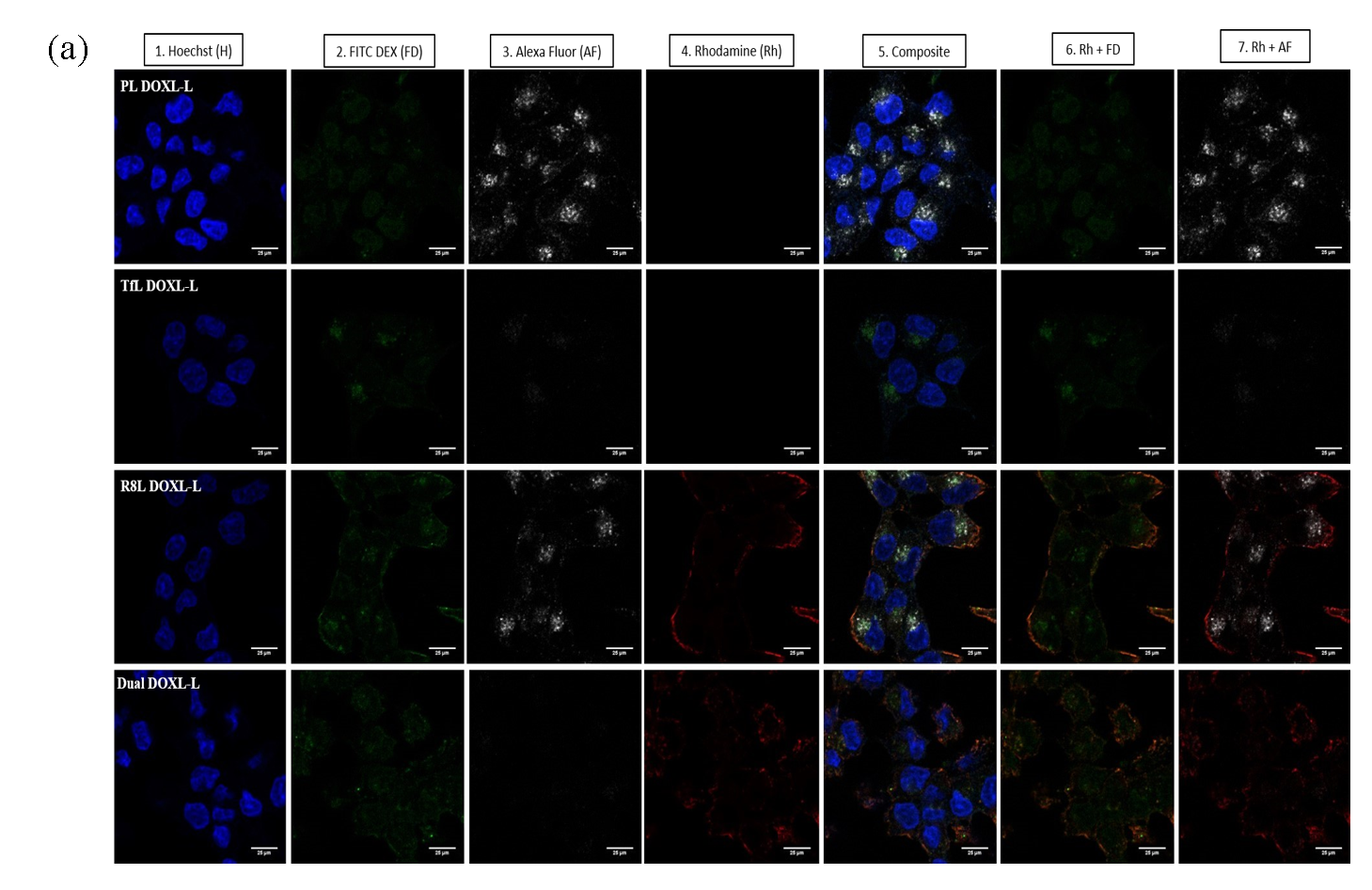


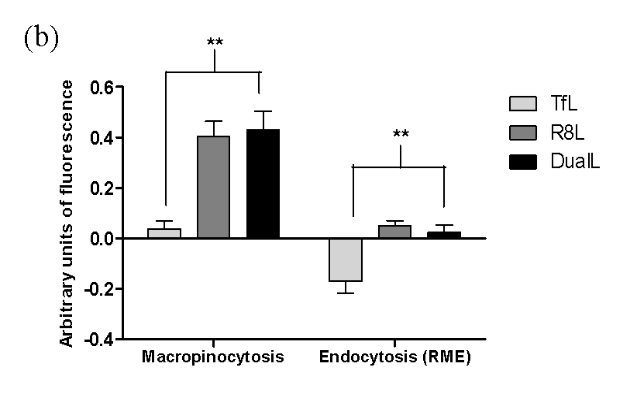

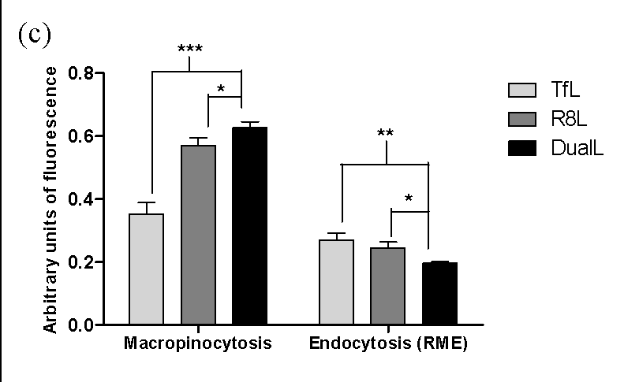


Supplementary Figure S6. RME and macropinocytosis evaluation to assay internalization of DualL.

(a.) A2780 cells were incubated with rhodamine-labelled PL,Tfl, R8L and DualL. Liposomes were added at a total lipid concentration of 0.1 mg/ml for a 4 h treatment period followed by analysis by confocal microscopy. (1) Nuclei stained by Hoechst 33342 at 5 µg/mL for 15 mins; (2) Macropinosomes stained by FITC Dextran 70 KDa at 0.35 mg /mL for 30 mins before formulation incubation; (3) Endosomes stained with Transferrin-Alexa fluor 680 at 10 µg /mL for 15 mins; (4) Rhodamine-signal from liposomes; (5) Merged composite picture of all the fluorescence; (6) Co-localization of rhodamine with FITC Dextran (7) Co-localization of rhodamine with Alexa Fluor. Yellow signals in the merged images indicate the co-localization of the red and green, red and pinkish fluorescence represents co-localization of red and gray, respectively. Analysis of fluorescence intensity-colocalization (Pearson's coefficient (b.) and Mander’s co-efficients (c.), obtained from the merged pictures (n=3) from TfL, R8L and DualL-treated cells, by Image *J* software. The results are mean ± SD averaged from three images of the same treatment. In figures *, **, ***, **** indicated p values ≤ 0.05, 0.01, 0.001 and 0.0001 respectively. Analyzed by Student’s t test. Scale bar, 25 µm.


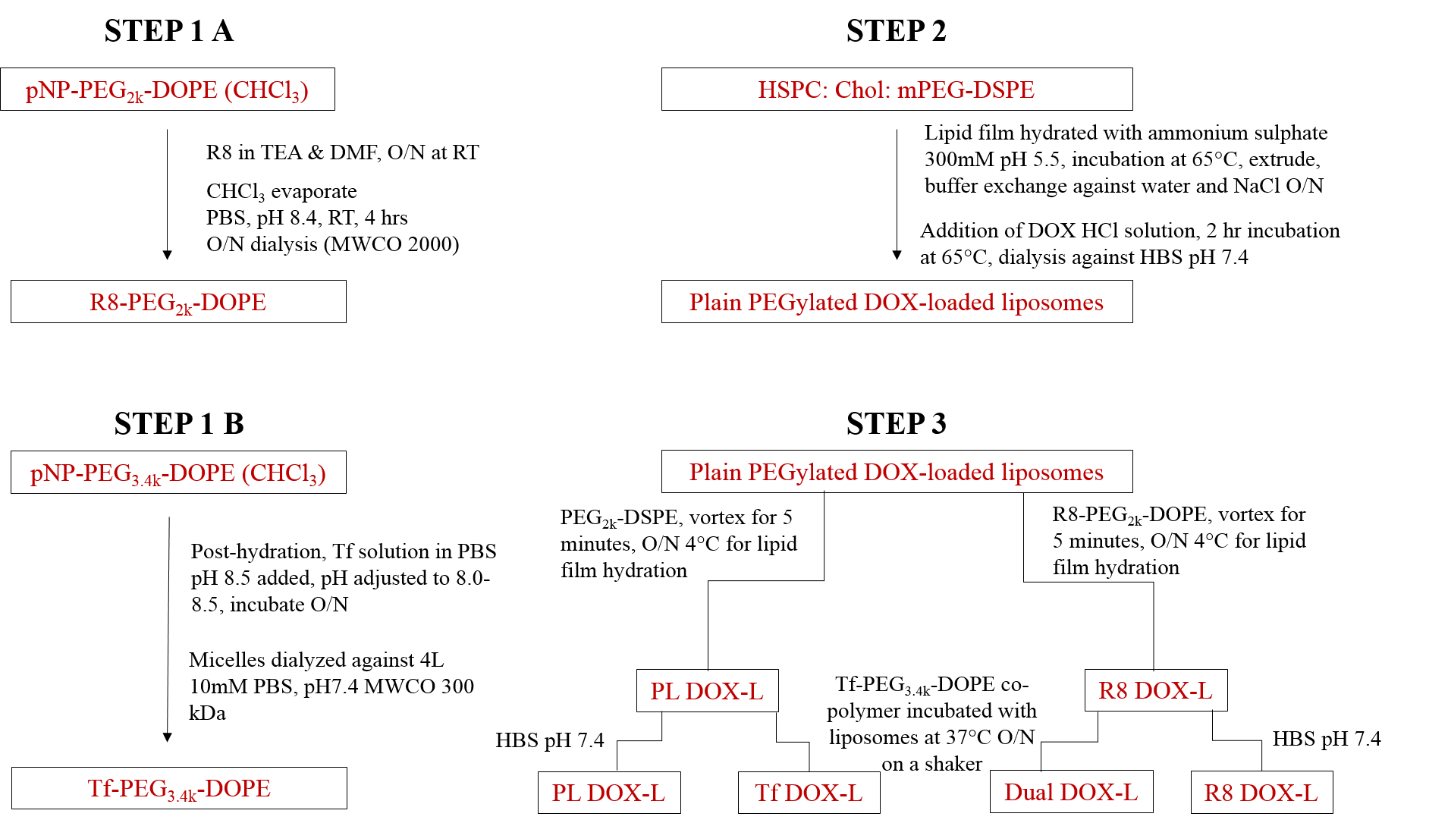


**Supplementary Figure S7.** Schematic representation of synthesis of single and dual ligand modified DOX-loaded liposomes.

| Liposomes (Abbreviation) | Composition of liposomes (mol %) | | | PEG_2000_-DSPE  (mol %) | R8-PEG_2000_-PE  (mol %) | Tf-PEG_3400_-PE (mol %) |
| --- | --- | --- | --- | --- | --- | --- |
|  | HSPC | Chol | mPEG-DSPE |  |  |  |
| PL DOX-L | 60 | 38.21 | 2 | 2 | - | - |
| R8 DOX-L | 60 | 38.21 | 2 | - | 2 | - |
| Tf DOX-L | 60 | 38.21 | 2 | 2 | - | 0.5 |
| Dual DOX-L | 60 | 38.21 | 2 | - | 2 | 0.5 |

**Supplementary Table 3.** Lipid composition of DOX-loaded liposomes

| **Sample** | **Mean diameter (nm)** | | **PDI** | | **zeta potential (mv)** | |
| --- | --- | --- | --- | --- | --- | --- |
|  | **Day1** | **Day 15** | **Day1** | **Day 15** | **Day1** | **Day 15** |
| **PL DOXL** | 203.0 ± 6.7 | 202.0 ± 6.3 | 0.1 ± 0.1 | 0.1 ± 0.1 | -32.0 ± 2.8 | -28.59 ± 3.8 |
| **TfL DOXL** | 220.6 ± 2.3 | 217.8 ± 11.9 | 0.2 ± 0.0 | 0.2 ± 0.1 | -31.1± 1.9 | -31.61 ± 4.0 |
| **R8L DOXL** | 210.6 ± 10.2 | 227.6 ± 26.2 | 0.2 ± 0.1 | 0.2 ± 0.0 | 9.9 ± 1.4 | 7.87 ± 5.2 |
| **DualL DOXL** | 230.9 ± 15.2 | 233.2 ± 27.7 | 0.2 ± 0.0 | 0.3 ± 0.1 | -20.2 ± 4.7 | -24.56 ± 2.2 |

**Supplementary Table 4.** Stability of DOX-loaded modified and unmodified liposomes at 4°C.


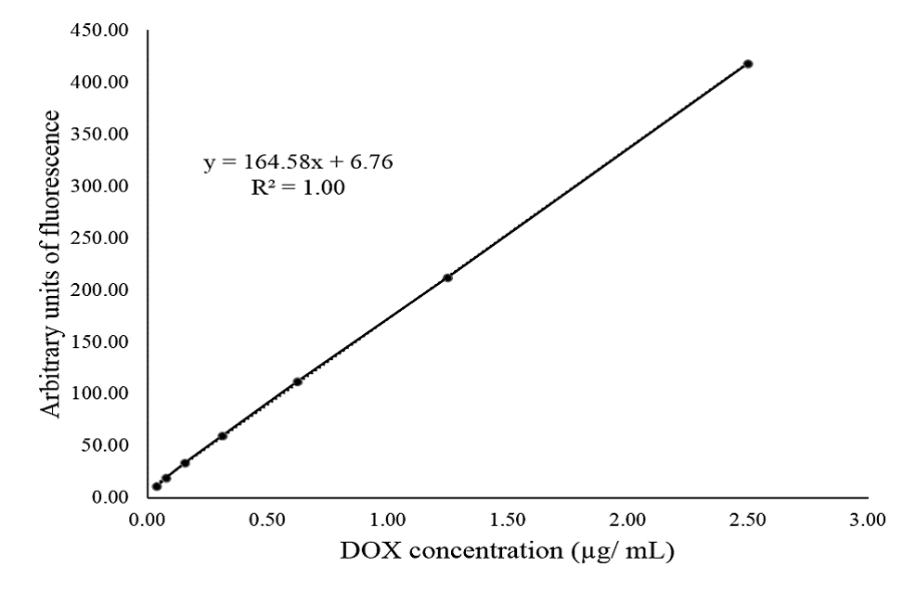


**Supplementary Figure S8.** Standard curve of free drug.

Standard curve for free Doxorubicin used to calculate the amount of drug encapsulated inside the liposomes


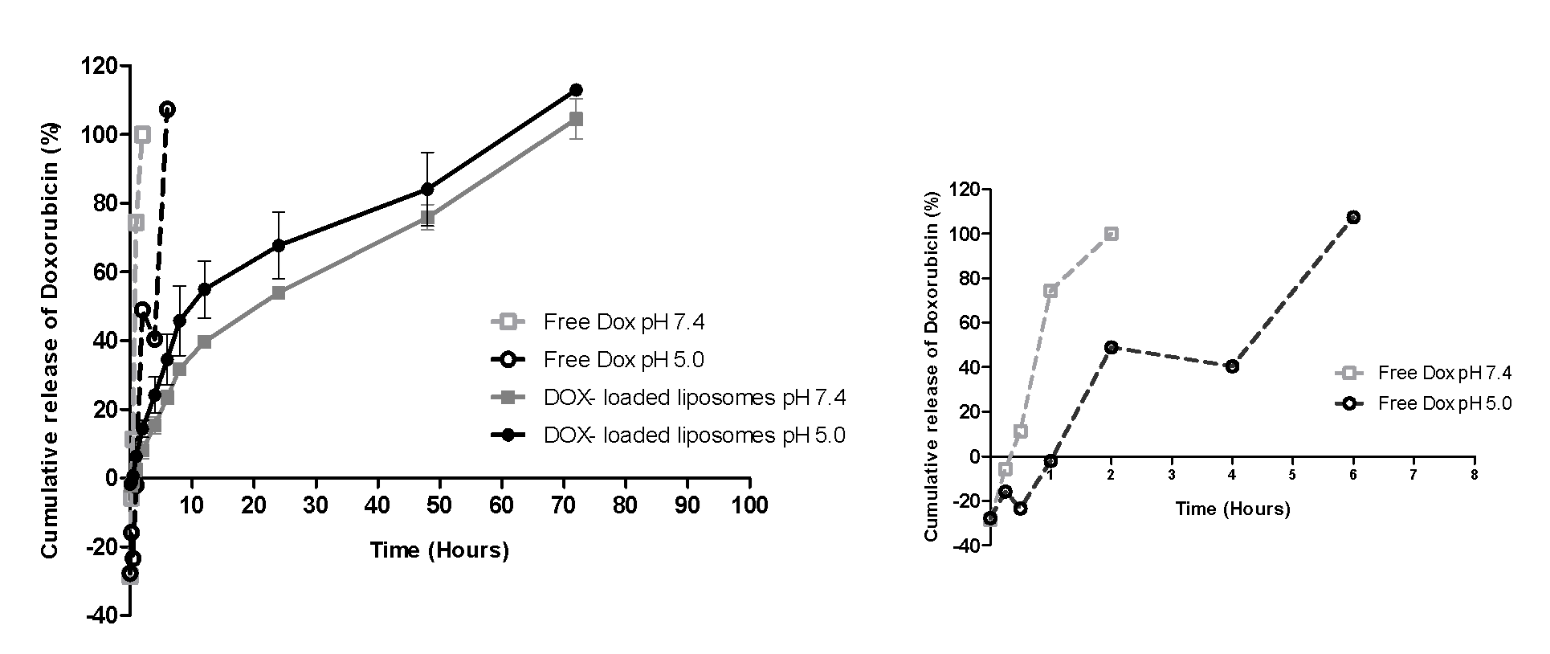


**Supplementary Figure S9.** Doxorubicin release profile of DOX-loaded liposomes *in vitro*.

The DOX release profiles from DOX-loaded PEGylated liposomes were studied in a time-course and pH-dependent release study at 37 °C. Free Doxorubicin was also tested under same conditions to test efficiency of the system. Results obtained by mean ± SD from different experiments.


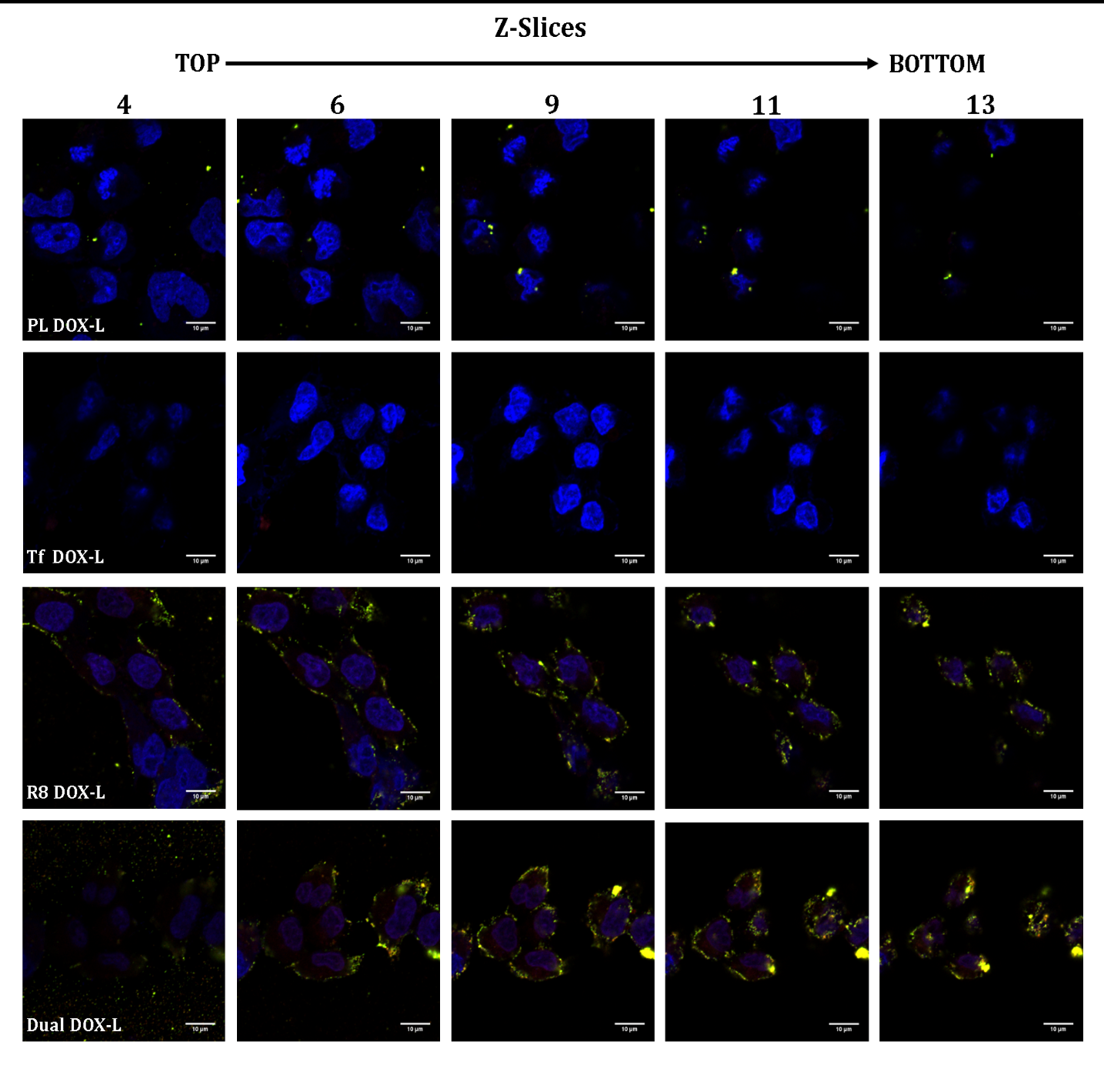


**Supplementary Figure S10.** Confocal microscopy- Z-stack images for DOX-loaded DiO-labelled liposomes

A2780 cells were incubated with Dio-labelled PL DOX-L, Tf DOX-L, R8 DOX-L or Dual DOX-L. Liposomes were added at a total lipid concentration of 0.1 mg/ml for 4 h treatment period followed by analysis by confocal microscopy. Yellow signals in the merged images indicate the co-localization of the red and green indicating cytoplasmic delivery, and purple fluorescence represents co-localization of red and blue indicating nuclear delivery, respectively. Scale bar, 10 µm.


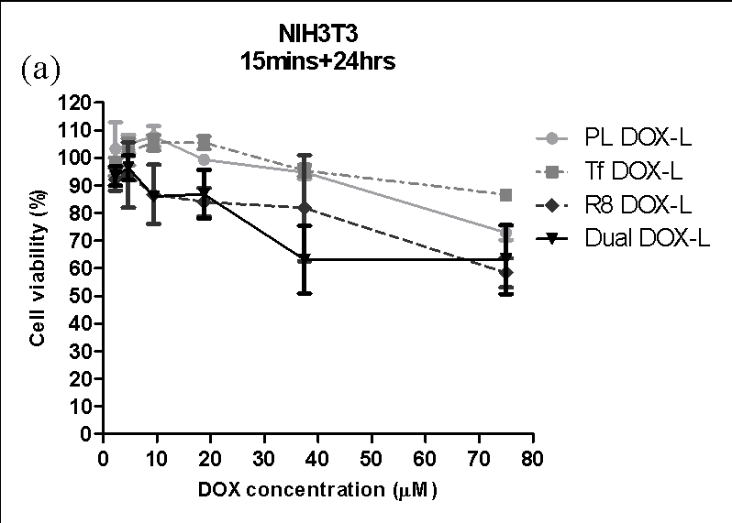
**
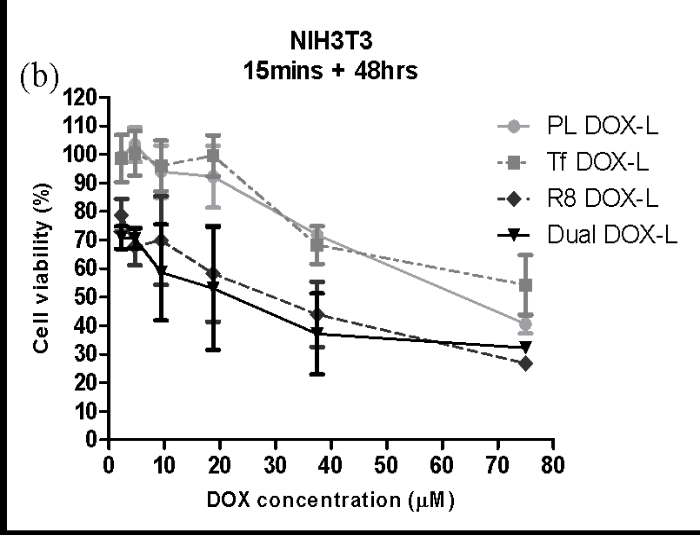
**


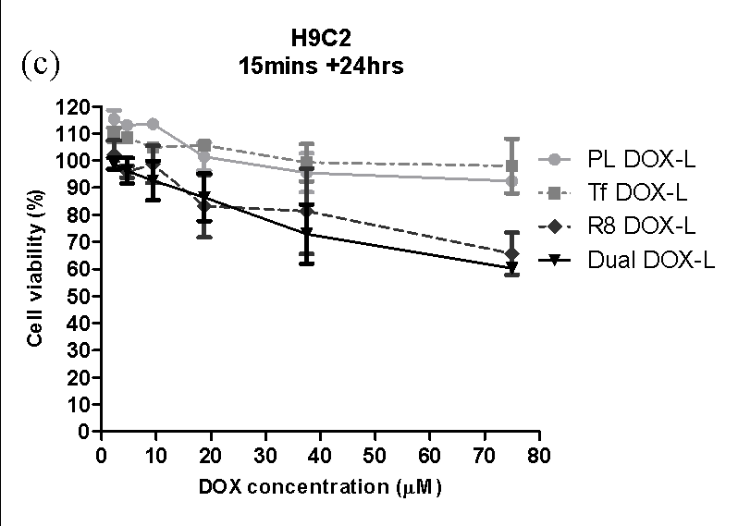

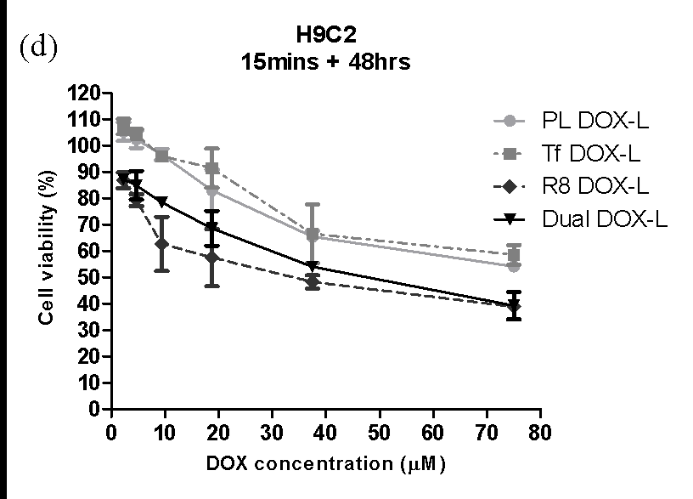

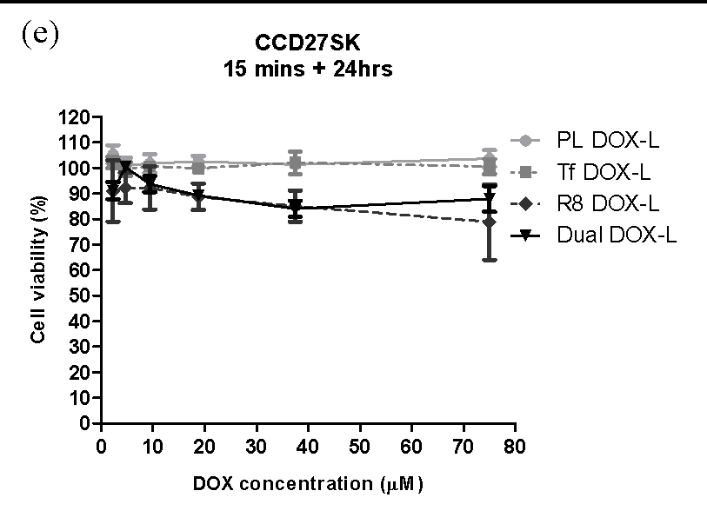

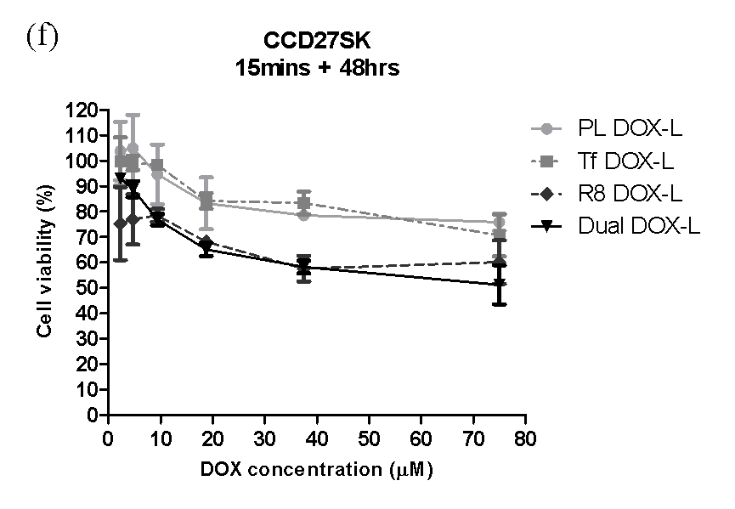


Supplementary Figure S11. Effect of dual functional DOX-loaded liposomes on cell death in non-cancer cells that do not over-express TfRs

Assessment of cell viability of (a & b) NIH3T3, (c & d) H9C2 and (e & f) CCD27Sk cells treated with DOX-loaded PL DOX-L, Tf DOX-L, R8 DOX-L and Dual DOX-L, at DOX concentration of 0.2µM-75µM for 15 minutes followed by 24 or 48 h incubations. Results obtained as mean ± S.D. from 2-3 separate experiments. * indicates *p*<0.05, ** indicates *p*<0.01, *** indicates *p*<0.001analyzed by One-way ANOVA.


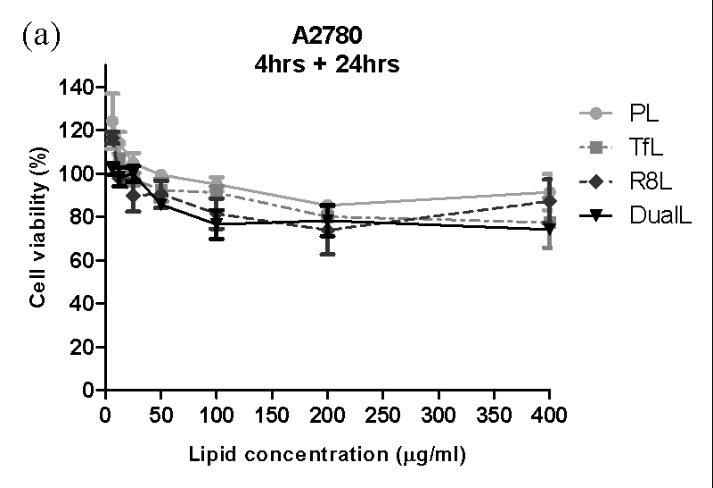

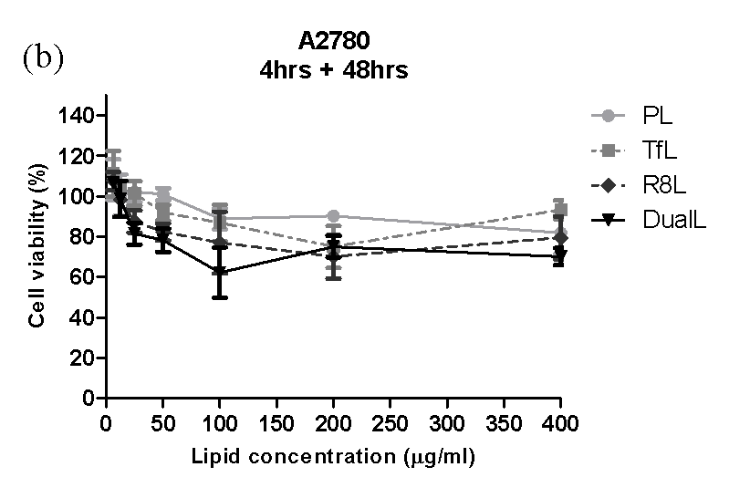


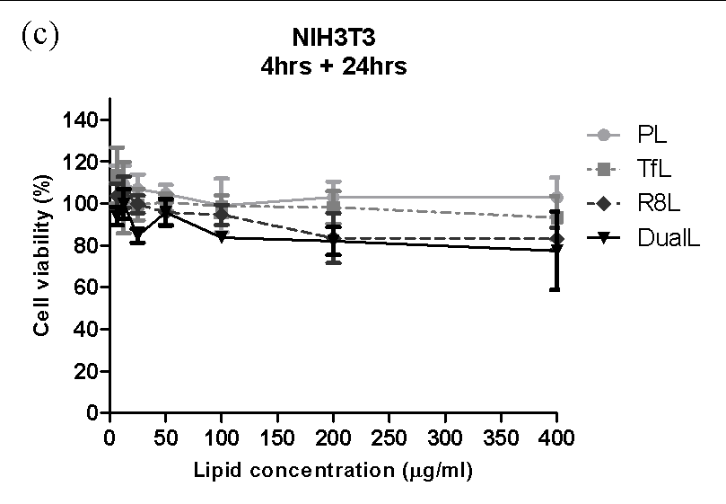

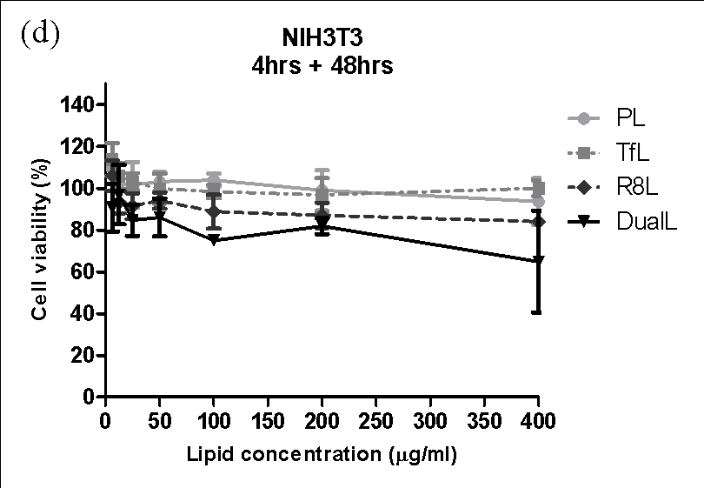


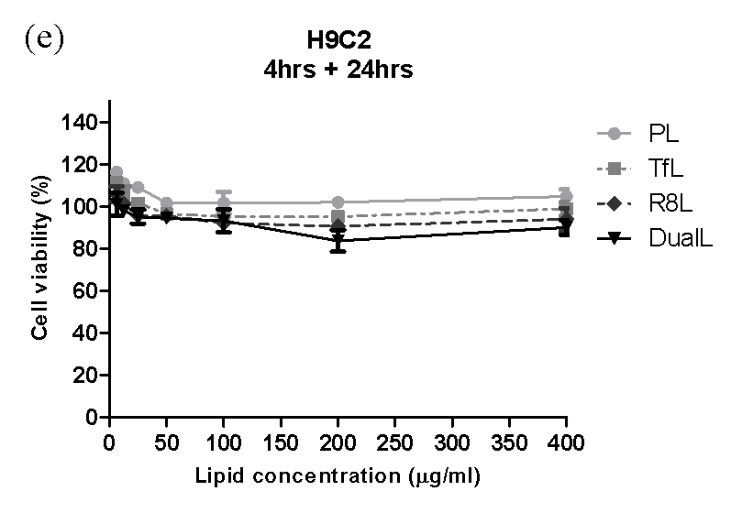

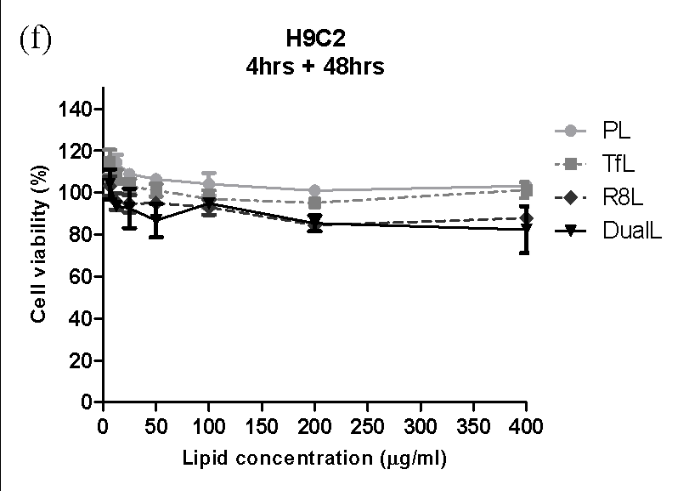


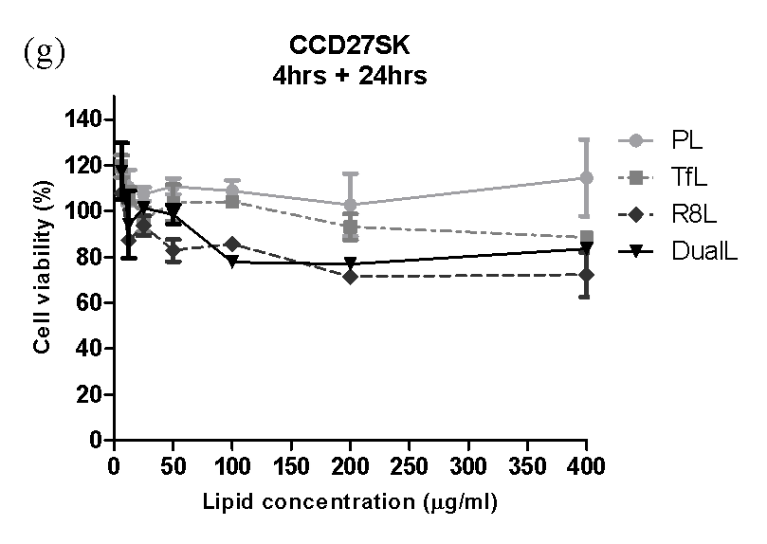

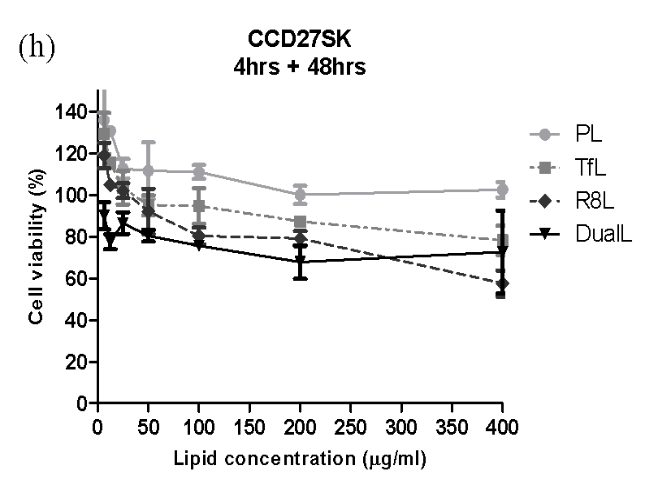


Supplementary Figure S12. Cytotoxicity of empty DOX-free liposomes.

Assessment of cell viability on (a & b) A2780 cancer cells; (c & d) NIH3T3; (e & f) H9C2 and (g & h) CCD27SK, with empty PL, TfL, R8L and DualL at lipid concentrations of 6-400 µg/mL was studied at a time-point of for 4 hours followed by 24 and 48 h incubation. The significance of difference between the means was analyzed by One-way ANOVA.


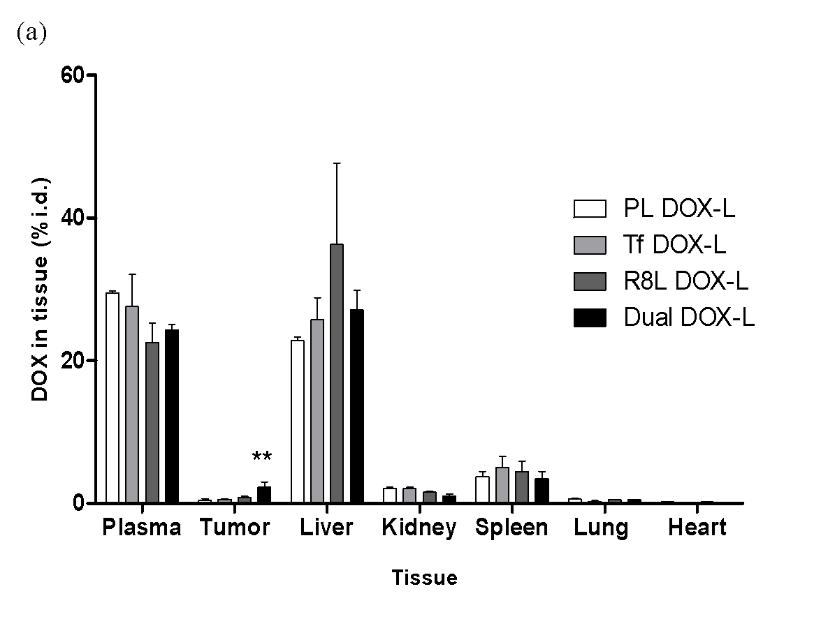

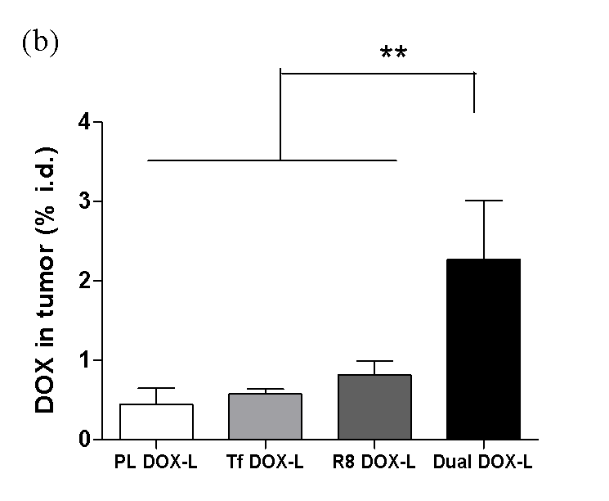


Supplementary Figure S13. Injected dose of Dox (%) in major organs of mice bearing A2780 tumors.

(a) Represents % injected dose of DOX in major organs. (b) Represents % injected dose of DOX in tumors across 4 tested groups. A single 10mg/kg i.v. tail injection of PL DOX-L, Tf DOX-L, R8 DOX-L or Dual DOX-L was administered. After 10 hours, the mice were sacrificed and major organs and tumors were collected. N= 2 animals per group ± SD. Analyzed by One way ANOVA where ***p* < 0.01.


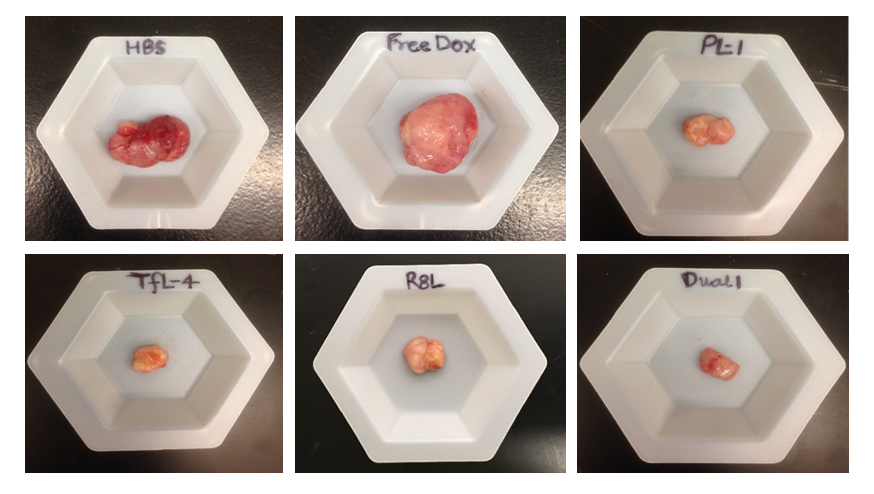


Supplementary Figure S14. Representation of A2780 tumors isolated at the end of the study.


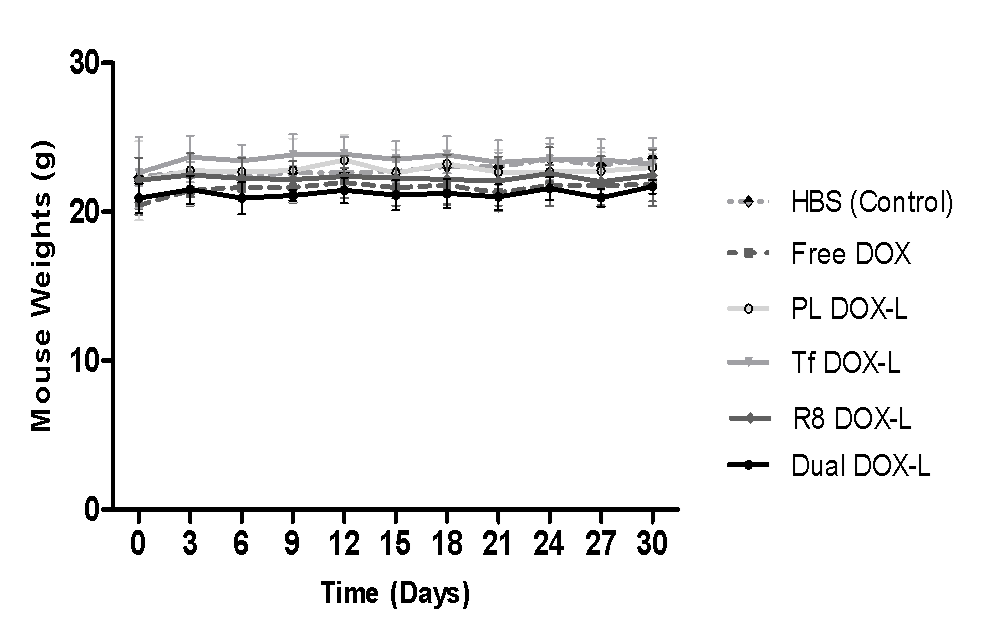


Supplementary Figure S15. Body weights of A2780 tumor bearing nude mice.

Body weights of A2780 tumor bearing nude mice throughout the treatment period. Data represented as mean ± SD.

Mice body weights were also observed and recorded throughout the study as an indicator of treatment toxicity. No significant differences were observed among mice treated with HBS and Free DOX versus all four treatment groups No differences (loss or gain in weight) in weights indicated minimum or no toxicity of formulations over the treatment period.
